# Reviewing the Prospective Pharmacological Potential of Isothiocyanates in Fight against Female-Specific Cancers

**DOI:** 10.3390/cancers15082390

**Published:** 2023-04-20

**Authors:** Shoaib Shoaib, Farheen Badrealam Khan, Meshari A. Alsharif, M. Shaheer Malik, Saleh A. Ahmed, Yahya F. Jamous, Shahab Uddin, Ching Siang Tan, Chrismawan Ardianto, Saba Tufail, Long Chiau Ming, Nabiha Yusuf, Najmul Islam

**Affiliations:** 1 Department of Biochemistry, Jawaharlal Nehru Medical College, Aligarh Muslim University, Aligarh 202002, India; 2 Department of Biology, College of Science, United Arab Emirates University, Al Ain 15551, United Arab Emirates; 3 Department of Chemistry, Faculty of Applied Sciences, Umm Al-Qura University, Makkah 21955, Saudi Arabia; 4 Department of Chemistry, Faculty of Applied Sciences, Assiut University, Assiut 71515, Egypt; 5 Vaccines and Bioprocessing Center, King Abdulaziz City for Science and Technology (KACST), Riyadh 12354, Saudi Arabia; 6 Translational Research Institute and Dermatology Institute, Academic Health System, Hamad Medical Corporation, Doha 3050, Qatar; 7 Laboratory of Animal Center, Qatar University, Doha 2731, Qatar; 8 School of Pharmacy, KPJ Healthcare University College, Nilai 71800, Malaysia; tcsiang@kpjuc.edu.my; 9 Department of Pharmacy Practice, Faculty of Pharmacy, Universitas Airlangga, Surabaya 60115, Indonesia; chrismawan-a@ff.unair.ac.id (C.A.); longchiauming@gmail.com (L.C.M.); 10 PAPRSB Institute of Health Sciences, Universiti Brunei Darussalam, Gadong BE1410, Brunei; 11 School of Medical and Life Sciences, Sunway University, Sunway City 47500, Malaysia; 12 Department of Dermatology, University of Alabama at Birmingham, Birmingham, AL 35294, USA

**Keywords:** Isothiocyanates, sulforaphane, benzyl isothiocyanate, phenethyl isothiocyanate, breast cancer, cervical cancer, ovarian cancer

## Abstract

**Simple Summary:**

Gynecological cancers are among the most commonly diagnosed cancers in women globally. Despite advancements in diagnostics and treatments, these cancers remain a significant health problem. Alternative medicines, such as isothiocyanates (ITCs), have gained attention for their potential effectiveness against various types of cancers. ITCs, including sulforaphane, benzyl isothiocyanate, and phenethyl isothiocyanate have demonstrated considerable ability to inhibit cancer cell growth, induce cell death, and modulate other cellular processes in female-specific cancers. Moreover, ITCs may enhance the chemo-sensitization of chemotherapeutic drugs, improving efficacy in combination with conventional treatments or other phytochemicals. Studies suggest that ITCs may be effective either as standalone treatments or in combination with conventional chemotherapies for the prevention or treatment of female-specific cancers. Improved understanding of the molecular intricacies of ITCs could lead to better treatment options for these cancers. Seemingly, a better understanding of these molecular aspects could open new horizons for ITC-based therapeutic interventions, potentially improving the prognosis of female-specific cancer patients.

**Abstract:**

Gynecological cancers are the most commonly diagnosed malignancies in females worldwide. Despite the advancement of diagnostic tools as well as the availability of various therapeutic interventions, the incidence and mortality of female-specific cancers is still a life-threatening issue, prevailing as one of the major health problems worldwide. Lately, alternative medicines have garnered immense attention as a therapeutic intervention against various types of cancers, seemingly because of their safety profiles and enhanced effectiveness. Isothiocyanates (ITCs), specifically sulforaphane, benzyl isothiocyanate, and phenethyl isothiocyanate, have shown an intriguing potential to actively contribute to cancer cell growth inhibition, apoptosis induction, epigenetic alterations, and modulation of autophagy and cancer stem cells in female-specific cancers. Additionally, it has been shown that ITCs plausibly enhance the chemo-sensitization of many chemotherapeutic drugs. To this end, evidence has shown enhanced efficacy in combinatorial regimens with conventional chemotherapeutic drugs and/or other phytochemicals. Reckoning with these, herein, we discuss the advances in the knowledge regarding the aspects highlighting the molecular intricacies of ITCs in female-specific cancers. In addition, we have also argued regarding the potential of ITCs either as solitary treatment or in a combinatorial therapeutic regimen for the prevention and/or treatment of female-specific cancers. Hopefully, this review will open new horizons for consideration of ITCs in therapeutic interventions that would undoubtedly improve the prognosis of the female-specific cancer clientele. Considering all these, it is reasonable to state that a better understanding of these molecular intricacies will plausibly provide a facile opportunity for treating these female-specific cancers.

## 1. Introduction

Cancer has emerged as a most notable health issue, which seems to be one of the primary reasons for exerting a social, psychological, and economic burden on the health system. In recent years, breast, ovarian, and cervical cancers have gained significant attention as they seem to be a global health problem affecting women, especially in middle- and low-income countries. Breast cancer is the most common, heterogeneous, and frequently diagnosed female-specific cancer, which is globally recognized as a major cause of cancer deaths among women. Of note, global estimates recorded 2.3 million women diagnosed with breast cancer and 0.685 million deaths in 2020 [1]. Epidemiological data indicate that at the end of 2020, there were basically 7.8 million women alive who were diagnosed with breast cancer in the previous 5 years, making it the world’s most prevalent cancer [1,2]. Accumulating evidence has suggested the involvement of various risk factors in the development of breast cancer. To this end, oral contraceptives [3], age [4], obesity [5], smoking [6], menopausal status [7], vitamin D deficiency [8], physical inactivity [9], marital status [10], a fat-rich diet [11], and alcohol consumption [12] are amongst others that have been recognized as the significant risk factors for breast cancer development. Furthermore, another well-known female malignancy is cervical cancer, which is primarily caused by high-risk human papillomaviruses, specifically HPV16 and HPV18. Epidemiological data suggest that more than 0.604 million new cases and 0.342 million deaths were reported in 2020 on a global scale [13,14]. Interestingly, almost 99% of cervical cancer patients were found infected with one of the HPV subtypes [15]. Moreover, it is evident from the epidemiological data that most of the affected women belong to less developed countries, as 12% of the total reside therein [16]. The detection of cervical cancer mostly relies on screening methods such as cytological diagnosis, colposcopic analysis, and p16/Ki-67 staining [17,18]. Disease initiation and progression is a critically regulated process, as HPV infection is merely inadequate for cervical cancer development. Use of contraceptives [19], exposure to diethylstilbestrol [20], active and passive smoking [21], genetic predisposition [22], immune evasion [23], genetic alterations [24], co-infection with either HIV or chlamydia [25,26], sexual history, a nutrient-poor diet [27], and social inequality [28] are the major risk factors which may enhance the chance of developing cervical cancer [29]. Another female malignancy is ovarian cancer; numerous evidence-based observations have identified ovarian cancer as a group of several histological subtypes arising from the ovary and fallopian tube epithelium. Histologically and genetically, ovarian cancer is a diverse group of tumor upsetting thousands of lives yearly as it seems to be the fifth cause of cancer deaths worldwide [30]. Epidemiological data suggest that approximately 0.314 million new ovarian cancer cases and 0.207 million deaths were reported in 2020 on a global scale [31]. In the past few decades, ovarian cancer has emerged as one of the leading causes of death, representing all four histological subtypes, such as mucinous, serous, endometrioid, and adenocarcinoma [32]. The lack of effective screening and non-specific symptoms cause delayed diagnosis of ovarian cancer and these are attributed to the escalation in mortality [33]. Despite extensive research in developing an effective screening test to detect ovarian cancer, there is not much success [34]. Unfortunately, the five-year survival rate of advanced stage ovarian cancer is only between 5 and 30%, representing the failure of first line chemotherapy and relapse with drug resistance in advanced stage ovarian cancer [35].

## 2. General Characteristics, Sources, and Biological Importance of Isothiocyanates

In the last few decades, there has been a growing interest in utilizing the potential of various phytochemicals in the fight against various pathological conditions [36,37,38,39,40,41,42,43,44,45,46,47]. Interestingly, both in vitro and in vivo studies have enlightened their potential against many disease pathologies, including metabolic, inflammatory, neurodegenerative, cardiovascular diseases, cancer, and so on [48,49,50,51,52,53,54,55,56]. Furthermore, a considerable number of studies provided proof of the health benefits of various dietary phytochemicals [57,58]. Of note, it is reasonable to argue that over the last two decades, bioactive compounds have gained great attention as therapeutic interventions against diverse disease conditions, either as solitary treatment and/or in combinatorial treatment regimens [59,60]. Concomitantly, with tremendous efforts laid down for scientific verification of pharmacological actions of various phytochemicals, and verification/validation of the basis of the use of these phytochemicals in the treatment of diseased conditions, including cancer, it is envisaged that they are not far from bringing therapeutic revolution.

In general, bioactive phytochemicals can be classified into various groups, including organosulfurs, phenolics, alkaloids, terpenoids, chalcones, glycosides, coumarins, etc. ITCs are the products of enzymatic hydrolysis of glucosinolates present in plants of the Brassicaceae family, and as secondary metabolites, they have been identified for their wide range of biological properties, including anticarcinogenic, antimicrobial, antioxidant, anti-inflammatory, and so on [61,62,63]. Table 1 highlights the various ITCs, including Sulforaphane (SFN), Benzyl isothiocyanate (BITC), and Phenethyl isothiocyanate (PEITC), along with their structures and plant sources.

## 3. Impacts of Isothiocyanates on Female-Specific Cancers

**Figure 1:**
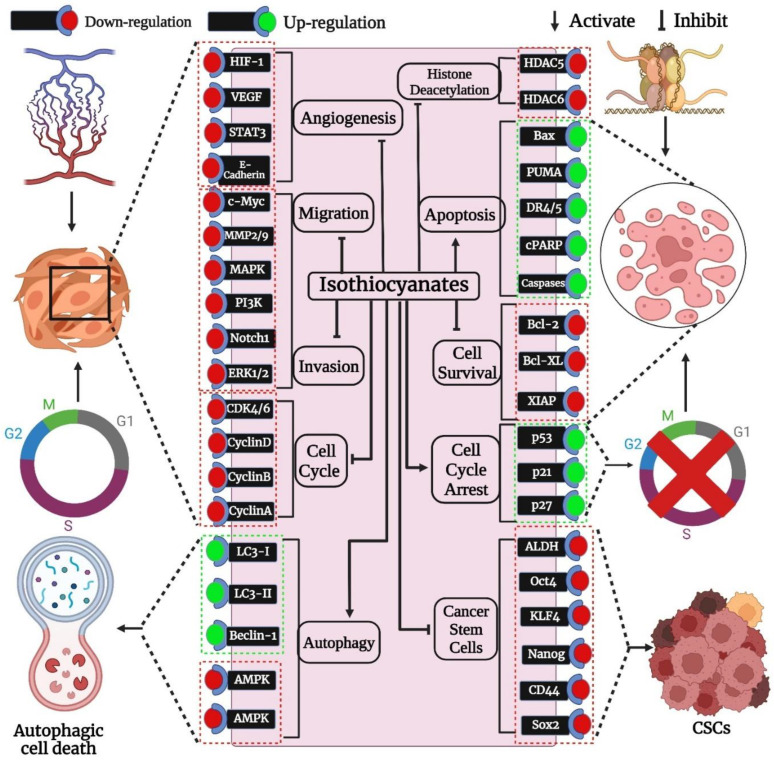
Representative figure highlighting the schematic illustration of the plausible mechanisms of action of ITCs against female-specific cancers. ITCs (SFN, BITC, and PEITC) effectively participate in inhibition of cell survival, migration, invasion, angiogenesis, and histone deacetylation, and lead to cell cycle arrest, apoptosis induction, and modulation of autophagy and cancer stem cells. The image was created in BioRender software (biorender.com).

Accumulating evidence indicates that cruciferous plants possess many bioactive phytochemicals which have been identified as potential chemopreventive agents [64]. In particular, cruciferous ITCs have been shown to considerably alter the cellular activities occurring during carcinogenesis [65]. Given that the bioactive phytochemicals, particularly ITCs play crucial role in modulation of molecular mediators of carcinogenesis [66], the extending data implies the importance of ITCs in the form of intriguing pharmacological interventions [67]. A significant number of reports have demonstrated that ITCs actively participate in modulation of various cellular processes, including the modulation of cancer cell proliferation, migration and invasion, cell cycle arrest, apoptosis induction, and modulation of autophagy and cancer stem cells (CSCs) [68]. Taking these into consideration, in this review, the advancement in knowledge regarding the aspects highlighting the molecular intricacies of ITCs in female-specific cancers has been discussed. In addition, we have also argued regarding the potential of ITCs either as solitary treatment or in combinatorial therapeutic regimen for the prevention and/or treatment of female-specific cancers. Hopefully, this review will open new horizons for consideration of ITCs in therapeutic interventions that would undoubtedly improve the prognosis of female clienteles. Collectively, it is reasonable to state that a better understanding of these molecular intricacies will plausibly provide a facile opportunity for treating female-specific cancers. Basically, for this review, we have followed the guidelines of the preferred reporting system for systematic reviews and meta-analysis (PRISMA). Different scientific databases including PubMed, Web of Science, Science Direct, Scopus, and Google Scholar were used to search the literature related to in vitro, in vivo, and human studies on anticancer potentials of respective ITCs [69]. Figure 1 highlights the molecular intricacies of the potential role of ITCs, whereas the studies highlighting the prospective role of ITCs against female-specific cancers have been collated in Table 2.

### 3.1. Impacts of Isothiocyanates on Cancer Proliferation, Migration, and Invasion-Related Signal Transduction Pathways

As a matter of fact, human cancers are basically characterized by frequent abnormalities in the signal transduction pathways that regulate cellular proliferation and survival. As a matter of fact, one of the oncogenic signal transduction pathways is PI3K/Akt/mTOR/S6K1, a pro-survival signaling pathway, often found to be hyperactive due to constitutive activation of PI3K or Akt and/or loss of PTEN (a negative regulator of the PI3K/Akt/mTOR/S6K1 pathway) [116,117]. Basically, the PI3K/Akt/mTOR/S6K1 pathway mostly contributes to uncontrolled cell proliferation, migration, neovascularization, and evasion of apoptosis, and occasionally mutations in Akt may also lead to chemoresistance.

#### 3.1.1. Impacts of SFN on Cancer Proliferation, Migration, and Invasion-Related Signal Transduction Pathways

Interestingly, molecular studies have demonstrated that SFN considerably inhibits the survival of MCF-7, MDA-MB-231, MDA-MB-238, and SKBR-3 breast cancer cells in a dose-dependent manner, seemingly through attenuation of phosphorylation of Akt and S6K1 [70]. Furthermore, it was found that SFN significantly inhibited the growth, proliferation, and colony formation in MCF-7 and MD-MB-231 cells, without having any effects on the non-tumorigenic cells viz MCF-10A [70]. Moreover, as a matter of fact, DNA methyltransferases (DNMTs) which are required for DNA methylation, play an important role in the normal development of mammalian cells plausibly through the maintenance of genomic stability. However, aberrant DNA methylation, seemingly due to hyper-activation of the DNMTs, has been closely linked with many cancers. To this end, it was found that SFN treatment seemingly leads to suppression of human telomerase reverse transcriptase (hTERT) expression and DNA methyltransferases (DNMT1 and DNMT3a) that correlates with demethylation of the exon 1 region of the hTERT promoter and the binding of a transcription factor, CTCF to hTERT, leading to repression of hTERT transcription in MCF-7 and MDA-MB-231 cells [71]. Concomitantly, SFN-treated MCF-7 and MDA-MB-231 cells corroborated decreased viability and proliferation inhibition in a dose- and time-dependent manner [73]. Another study also showed that SFN conferred cytotoxicity on breast cancer cells (MCF-7, MDA-MB-231, MDA-MB-468, and T47D) in a time- and dose-dependent manner [74]. Furthermore, transcriptional regulation of hTERT is considered to activate telomerase in human cancers, which maintains or lengthens telomeres, and hTERT up-regulation is mostly achieved by genetic and epigenetic alterations. To this end, it has been found that SFN treatment inhibited hTERT in a dose- and time-dependent manner [71]. Furthermore, the balance between the activity of histone acetylation and deacetylation, which is achieved by histone acetyl transferases (HATs) and histone deacetylases (HDAC), is very critical for the normal functioning of the cells. However, variations in the expression of HDACs or mutations in their genes have been correlated with the development of different cancers [118]. Interestingly it was reported that SFN treatment leads to the inhibition of global HDAC activity, as also discussed below [74]. Furthermore, studies have shown that SFN triggered both single- and double-stranded breaks, leading to elevated phosphorylation of Ataxia-telangiectasia mutated (ATM), a protein kinase regulating DNA damage responses, and its expression results in the suppression of cancer growth, migration, and invasion [76,119,120]. Intriguingly, it is widely accepted that the cross-talk between histone deacetylase (HDACs) and lysine-specific demethylase 1 (LSD1) facilitates cancer cell growth and proliferation, and interestingly, it was demonstrated to be prevented following SFN treatment in MDA-MB-231 cells [77].

Furthermore, it has been highlighted that SFN treatment considerably suppressed the dynamic instability behavior of purified microtubules, which indicates that SFN-mediated cytotoxic effects on breast cancer cells were due to direct action on the microtubules as well [121]. Another study was conducted on KPL-1 breast cancer cells, wherein SFN treatment resulted in the growth inhibition of these cells in a dose- and time-dependent manner. Additionally, it suppressed the growth and metastasis of orthotopically transplanted KPL-1 cells in female athymic mice [122]. Furthermore, a study highlighted that SFN considerably suppressed proliferation of MCF-7 cells at IC50 of 12.5 and 7.5 µmol following 24 h and 48 h treatment, respectively, and this aspect was linked with the inhibition of estrogen receptor alpha protein together with inhibition of progesterone receptor [72].

Moreover, SFN-treated MDA-MB-231, MCF-7, and SKBR-3 cells exhibited a decrement in the viability of metabolically active cells in a dose-dependent manner, whereas no such effects were observed on HMEC normal cells [76]. The key cellular regulator responsible for the SFN cytotoxic effects was the induction of oxidative and nitrosative stress; since SFN treatment led to intracellular and mitochondrial ROS generation, protein carbonylation, and nitric oxide production in all breast cancer cell lines [76].

As a matter of fact, Wnt/β-catenin pathway activation is associated with the regulation of embryonic development and other physiological processes, while aberrant activation of this pathway results in the increased expression of β-catenin protein that augments metastasis, self-renewal of cancer stem cells (CSCs), chemoresistance, neovascularization, and immune evasion [123]. To this end, one of the studies showed that SFN down-regulated the Wnt/β-catenin pathway, resulting in considerable inhibition in cell viability and apoptosis induction in MCF-7 and SUM159 cells [75].

Over the years, studies have shown that SFN significantly exerted cytotoxic effects on cervical cancer cells (HeLa, Cx, and CxWJ) seemingly by promoting proliferation inhibition [84]. SFN induced growth inhibition in HeLa cells in a dose-dependent manner through apoptosis induction and reduction in inflammation-related proteins [84]. Another study found that SFN modifies epigenetic events causing cervical cancer. It was found that SFN reactivates tumor suppressor genes (TSGs) via inhibition of HDAC1 and DNA methyltransferase (DNMT3B) in human cervical cancer cells (HeLa) [85]. SFN has been shown to inhibit HeLa cell viability by apoptosis induction, as observed by the formation of apoptotic bodies and an increase in the sub-G1 cells [86]. Likewise, another interesting study reported that SFN exerts dose-dependent cytotoxicity against HeLa cells mediated by apoptosis and the anti-phlogistic effect [124].

Uncontrolled growth of cancer cells results in hypoxic conditions leading to the activation and transport of hypoxia-inducible factor-1α (HIF-1α) into the nucleus from the cytosol, wherein it acts as a transcription factor and promotes the expression of genes encoding for glucose transporter and angiogenesis-related proteins that influence glycolytic metabolism, cell growth, survival and angiogenesis thereof [125]. To this end, an in vitro study highlighted that SFN decreased the expression of HIF-1α and GLUT-1, suggesting SFN can efficiently inhibit HIF-1-mediated proliferation and migration in A2780 and A2780/CP ovarian carcinoma cells [83]. Moreover, the role of oncogenic c-Myc (transcription factor) has been investigated in many cancers, and in particular, oncogenic expression of c-Myc is activated by aberrant upstream signaling, super-enhancer activation, chromosomal amplification, and translocation, resulting in cell proliferation and chemoresistance. Targeting the expression of c-Myc may lead to considerable inhibition of cancer cell proliferation and enhanced sensitization of cancer drugs to chemoresistant cells [126]. Previously, one study highlighted that SFN considerably inhibits OVCAR-3 and A2780 cell growth, proliferation, colony formation, and metastasis. Further exploration of the mode of action highlighted that SFN-mediated cytotoxicity occurred in response to the suppression of c-Myc along with the inhibition of Akt and NF-κB thereof [80]. SFN-exposed SKOV-3 and OVCAR-3 cells demonstrated concentration-dependent decrement in the cell density following 48 h of exposure, seemingly through induction of apoptosis [127]. Moreover, dose- and time-dependent anti-proliferative effects of SFN were observed at IC50 of 40 µmol/L for SKOV-3, and 25 µmol/L for C3 and T3 cells, respectively [39]. SFN was very effective in inhibiting the clonogenicity of these ovarian cancer cells and also caused nuclear fragmentation and cellular morphological changes in SKOV-3, C3, and T3 cells [39]. Another in vitro study indicated that SFN inhibited PA-1 ovarian cancer cells in a dose- and time-dependent manner [81]. Moreover, SFN decreased the viability of MDAH2-774 and SKOV-3 ovarian cancer cells in a time- and dose-dependent manner, and the study highlighted considerable cellular morphological changes in these treated cells [82]. Of note, NF-κB is a transcription factor that has been studied extensively, and the emerging data indicate that NF-κB has multiple activators, including epidermal growth factor receptor (EGFR) and TNF-α. Evidence has shown that the activation of NF-κB has been linked with cancer cell proliferation, survival, metastasis, inflammation, neovascularization, and chemo- and radioresistance [128,129]. These aspects highlight that NF-κB could be a valuable pharmacological target for therapeutic intervention against cancer. Different points in the NF-κB pathway have been targeted to inhibit and/or regulate NF-κB activation. In the past few years, much effort has been devoted to the development and characterization of NF-κB blocking agents, including natural as well as synthetic compounds. A significant amount of progress has been made in the preclinical and clinical studies, and some anticancer compounds with NF-κB-inhibiting properties, such as bortezomib, are already being used clinically. Interestingly, but not surprisingly, it has been highlighted that SFN can strongly inhibit the NF-κB signaling pathway in breast carcinoma cells and considerably attenuates 12-O-tetradecanoyl phorbol-13-acetate (TPA)-induced Matrix metalloproteases (MMP-9) expression thereof. Collectively, the study explicitly demonstrated that SFN significantly suppresses TPA-stimulated cancer cell invasion; thus, it could be a prospective candidate for the development of intriguing therapeutics for the prevention of breast tumor invasion and metastasis in the in vivo model [78].

#### 3.1.2. Impacts of BITC on Cancer Proliferation, Migration, and Invasion-Related Signal Transduction Pathways

Over the years, studies have shown that BITC suppressed the growth of MCF-7 and MDA-MB-231 cells through apoptosis induction and cell cycle arrest [88]. The altered expression of EMT-associated proteins, the increased expression of E-cadherin, and occludin with concomitant down-regulation of Snail, vimentin, fibronectin, and c-Met supported the declined epithelial–mesenchymal transition (EMT) potential [89]. An in vitro and in vivo study also indicated that BITC efficiently inhibited EMT, migration, and invasion, owing to repression of urokinase-type plasminogen activator (uPA) in breast cancer cells [90]. In an in vitro study, in addition to DNA fragmentation, the number of Annexin-V and propidium iodide-positive cells was demonstrated to be increased following treatment with BITC, indicating apoptosis induction in HeLa cells, which was further correlated with the decreased ATP level [98]. The mechanistic insights highlighted that BITC treatment resulted in the increased production of ROS, which led to caspase-3 activation in HeLa cells. BITC exposure to HeLa and MRC-5 cells decreased cell viability in a time- and dose-dependent manner [99]. Furthermore, BITC treatment was more toxic to the dividing cells, owing to reduced phosphorylation of Aurora A [99]. BITC-treated ovarian cancer cells undergo growth and proliferation inhibition in a dose- and time-dependent manner, seemingly due to the induction of DNA fragmentation, condensation, and eventually apoptosis induction [130]. As a matter of fact, Notch2 is a Notch receptor that is frequently overexpressed in a variety of malignancies and is associated with a distinct carcinogenic process. Evidence has shown that the Notch2 receptor has been targeted by several plant products, and one such study highlights that Notch2 activation by BITC impedes its inhibitory effect on breast cancer cell migration [94].

#### 3.1.3. Impacts of PEITC on Cancer Proliferation, Migration, and Invasion-Related Signal Transduction Pathways

It is widely acknowledged that neovascularization is a critical step in the progression of tumor growth and metastatic dissemination. As a result, the molecular basis of neovascularization has piqued the curiosity of cancer researchers. One of the primary regulators of this process is the vascular endothelial growth factor (VEGF) pathway. Once the VEGF-receptor pathway is triggered, it, in turn, activates multiple signal transduction pathways resulting in the promotion of endothelial cell proliferation, migration, and neovascularization [131]. Therefore, many of the bioactive phytocompounds have been explored in order to block aberrant expression of VEGF to control and prevent cancer. One such phytocompound is PEITC, which exerted cytotoxic effects primarily through the extensive generation of reactive oxygen species (ROS), leading to the decreased expression of hypoxia-sensitive HIF-1α protein and heat shock Hsp90 protein, which further attenuated the expression of MMP2, MMP9, and VEGF to attenuate cellular adhesion, migration, and invasion of MCF-7 and MDA-MB-231 cells [100]. Notably, the same study indicated that PEITC also enhanced nuclear accumulation of the nuclear factor erythroid2-related factor2 (Nrf2) that acts as a master switch to the regulation of redox homeostasis. Moreover, PEITC suppressed MDA-MB-231 and MCF-7 cells by inducing DNA fragmentation that resulted in apoptosis induction and cell cycle arrest thereof [101]. PEITC treatment suppressed cell viability of MDA-MB-231, T47D, BT549, MCF-7, SKBR3, and ZR-75-1 at IC50 concentrations of 7.2 µmol, 9.2 µmol, 11.9 µmol, 10.6 µmol, 26.4 µmol, and 40.4 µmol, respectively [132].

Oncogenic activation of mitogen-activated protein kinase/extracellular signal-regulated (MAPK/ERK) pathway may occur in response to the upstream genomic events as well as by activation of other multiple-associated signaling pathways. Especially, hyperactivation of MAPK/ERK1/2 mostly results in cancer development and progression as the pathway help in the survival, proliferation, and metastatic properties of cancer cells [133]. An in vitro study displayed that treating breast cancer cells with PEITC abrogated MAPK/ERK1/2 signaling pathway and down-regulated estrogen receptor (ER-α36), leading to the growth inhibition of breast cancer cells [102]. Furthermore, PEITC treatment resulted in the restoration of normal p53 checkpoint control pathway in mutant p53-expressing cancer cells, which normally functions to inhibit cell transformation, and its inactivation has been correlated with tumor cell growth and survival [134]. Moreover, PEITC exerted a significant cytotoxic effect on BRI-JM04 cells derived from a mammary tumor of an MMTV-neu transgenic mouse, seemingly through nuclear fragmentation and cleavage of poly (ADP-ribose) polymerase (PARP) [104]. Furthermore, PEITC down-regulated cadherin 1 by attenuating DNMT and HDAC activities, which further suppressed Wnt/β-catenin signaling, limiting colony formation and the growth of breast cancer cells [109].

Of note, CD44 hyperactivation has been reported in many cancers, and binding of CD44 with hyaluronan ligand results in oncogenic activation of signaling pathways which endorse induction of cell proliferation, migration, survival, EMT, and adaptive cancer plasticity [135]. One other such signaling molecule is ICAM1, which is localized at the cell surface as a receptor glycoprotein, and several studies have identified ICAM1 as a key signaling molecule contributing to cancer cell proliferation, migration, invasion, and neovascularization [136]. Thus, CD44 and ICAM1 both have been studied extensively as selective therapeutic targets in the field of cancer therapy and prevention. It has been envisaged that PEITC treatment resulted in the down-regulation of CD44, ICAM1, and also MMPs (MMP2 and MMP9), which further promoted suppression of invasion and migration of HeLa cells [109]. PEITC also showed significant growth inhibitory effects on cervical cancer cells by inducing apoptosis in HEp-2 and KB cervical cancer cells [108]. Of note, the transforming growth factor-β (TGF-β) is a receptor protein that has been documented to control several cellular processes, and TGF-β, upon binding with its ligand, results in the phosphorylation of SMADs; thereby, phosphorylated SMADs translocate to the nucleus to induce the transcription of several genes which foster oncogenic cellular processes including proliferation, evasion of apoptosis, EMT, and metastasis in late stages of cancer [137]. Interestingly, PEITC was reported to down-regulate TGF-β and Smad2 signaling, which accompanied alterations in the expression of metastasis-associated signaling molecules in HeLa cells [106]. Furthermore, another study demonstrated that PEITC exerted significant cytotoxic effects on Caski and HeLa cells in a dose- and time-dependent manner through the production of intracellular and mitochondrial ROS [138]. Additionally, the study also demonstrated that PEITC treatment caused significant alterations in the morphology of CaSki and HeLa cells, whereas not many significant alterations in the morphology of HaCaT cells (normal skin cell line) were observed [138].

Of note, signal transducers and activators of transcription (STAT3) are recognized as important regulators of various biological processes such as cell migration, survival, neovascularization, apoptosis, and cell cycle progression [139]. In this direction, numerous researchers have highlighted STAT3 as a potent therapeutic target for different phytocompounds such as polyphenols, organo-sulfur compounds, chalcones, etc. Previously, it has been envisaged that mTOR-STAT3 signaling was targeted by PEITC, which eventually led to cytotoxic effects on ovarian cancer cells [111]. Furthermore, PEITC-treated SKOV-3, OVCAR-3, and TOV-21G cells showed a significant decrement in the cellular proliferation in a dose-dependent manner following 24 h of exposure, and the IC50 for SKOV-3, OVCAR-3, and TOV-21G cells were found to be 15 µmol, 20 µmol, and 05 µmol, respectively [113]. PEITC treatment reduced the growth of SKOV-3, OVCAR-3, and NUTU-19 cells in a dose-dependent manner with IC50 values around 27.7 µmol, 23.2 µmol, and 25.1 µmol for SKOV-3, OVCAR-3, and NUTU-19 cells, respectively [87]. Furthermore, PEITC significantly inhibited migration and invasion properties of ovarian cancer cells (SKOV-3 and HO8910) and suppressed metastatic properties of epithelial ovarian cancer in a dose-dependent manner [111].

It is widely acknowledged that in the course of metastasis, metastatic cells detach from the primary tumors, enter the blood circulation by invading through stromal tissues, and thereby form metastatic colonies by invading the target organ. Of note, metastasis is one of the major hallmarks of cancer cells, in which numerous signaling molecules contribute to the invasion and migration of tumor cells. Invasion of a cancer cell is promoted by MMPs, zinc-dependent endopeptidases which disassemble the extracellular matrix (ECM). Accumulating evidence has shown that increased activities of MMPs (MMP2, MMP7, and MMP9) play an important role in cell proliferation, survival, invasion, and angiogenesis [140,141]. Interestingly, evidence has shown that ITCs regulate various signal transduction pathways and considerably inhibit the expression/activity of MMPs [142,143,144].

The aforementioned data explicitly highlight the importance of ITCs in the modulation of female-specific cancer proliferation, migration, and invasion-related signal transduction pathways through various intricate mechanisms.

### 3.2. Impacts of Isothiocyanates on Modulation of Cell Cycle

As a matter of fact, the eukaryotic cell cycle is a tightly regulated process in which a cell undergoes a series of coordinated events, including cell growth, DNA synthesis, and division; perhaps the cell cycle is preliminarily divided into G1, G2, S, and M-phase, and each phase is characterized by their respective cyclins and cyclin-dependent kinases (CDKs). Basically, cyclin D-CDK4/6, cyclin E-CDK2, cyclin A-CDK2, and cyclin B-CDK1 are restricted to G1 phase, G1-S transition, S-phase, G2-phase, and M-phase, respectively. Furthermore, cell cycle progression and arrest are both controlled by various cell cycle regulators. Intriguingly, in the last few decades, various phytochemicals have been demonstrated to block cell cycle progression through various intricate mechanisms, and in fact, lately, these phytochemicals have played an interesting role in controlling cancer.

#### 3.2.1. Impacts of SFN on Modulation of Cell Cycle

It has been envisaged that SFN treatment effectively hampered cell cycle progression in MCF-7 and MDA-MB-231 cells seemingly through the down-regulation of cyclin A, cyclin B, and CDC2; however, at the same time, a significant increment was observed in the expression of p21 and p27, which are the key cell cycle regulators [73]. Furthermore, SFN treatment effectively inhibited cell growth, induced a G2 m cell cycle block, and increased expression of cyclin B1 in MDA-MB-231, MDA-MB-468, MCF-7, and T47D cells [74]. Collectively, the study highlighted that SFN inhibited cell growth, activated apoptosis, inhibited HDAC activity, and decreased the expression of key proteins involved in breast cancer proliferation [74]. Furthermore, SFN treatment of MDA-MB-231, MCF-7, and SKBR-3 cells increased the percentage of cells in G0/G1, while the compound reduced the percentage of cells in S and G2/M. The mechanism of action of SFN was revealed to hamper the cell cycle progression by elevating the expression of cell cycle regulator proteins such as p53, p21, and p27 [76]. SFN-induced cell cycle progression inhibition was linked with reduced Akt signaling, global DNA hypomethylation, and genomic instability [76]. Additionally, SFN reduced the percentage of cells in the G1 and S phases while elevating the percentage of cells in G2/M progression in MCF-7 cells; however, the study did not indicate the mode of action of SFN to block cell cycle [121].

Furthermore, SFN-mediated cytotoxic effects on HeLa, Cx, and CxWJ were seemingly envisaged to the modulation of cyclin B1 and GADD45β, thereby leading to G2/M cell cycle arrest [84]. SFN treatment also induced cell cycle arrest by epigenetic modulations of DNMT3b [85], as also highlighted in our recent review [69]. Furthermore, SFN treatment resulted in a significant decrease in the percentage of cells in the S and G2/M phases of the cell cycle, while there was a significant increment in the percentage of cells in the G1 phase, indicating SFN-induced G1 cell cycle arrest in SKOV-3 and OVCAR-3 cells [127]. Furthermore, another in vitro study displayed that SFN caused cell cycle arrest in a dose- and time-dependent manner seemingly by reducing the expression of cell cycle-related proteins such as cyclin D1, CDK4, and CDK6 in SKOV-3, T3, and C3 ovarian cancer cells [39]. Mechanistic insights revealed that SFN suppressed the expression of Akt, PI3K, and GSK3-β, indicating the involvement of the Akt-PI3K signaling pathway in cancer initiation and progression [39]. SFN exposure significantly increased the percentage of cells in the G2/M phase while it decreased the percentage of cells in the G1 and S phases in a concentration-dependent manner, indicating SFN caused G2/M cell cycle arrest by down-regulation and dissociation of cyclin B1/Cdc2 complex in PA-1 cells [81]. Moreover, SFN-treated MDAH-2774 cells undergo G1 cell cycle arrest, which was clearly evident from the flow cytometry data, wherein the percentage of cells in the G0/G1 phase increased in comparison to the vehicle control, while there was a negligible increment in the percentage of cells in S phase [82]. Overall, there was a 1.5, 1.0, and 1.5 fold decrement in the expression of retinoblastoma (RB), E2F-1, and E2F-2 proteins, respectively, in SFN-exposed MDAH-2774 cells. Additionally, there was a reduction in the expression of CDK4 and CDK6, as revealed by Western blot analysis [82]. Furthermore, SFN-treated OVCAR-3 and A2780 cells undergo cell cycle arrest seemingly due to the altered expression of cell cycle-associated molecules, including cyclin D1, p27, and p53 [80].

#### 3.2.2. Impacts of BITC on Modulation of Cell Cycle

Of note, it has been envisaged that BITC treatment on MCF-7 and MDA-MB-231 cells showed cell cycle arrest, which occurred in response to the down-regulation of important cell cycle regulators such as cyclin B1, CDK1, and Cdc25C [88]. Amongst the various plausible reasons, BITC-mediated cytotoxicity on cervical cancer cells may be due to the perturbation in the cell cycle; interestingly, accumulation of cells in the G2 and M phase was observed in a dose- and time-dependent manner in both cell lines (HeLa and MRC-5) following treatment with BITC, which may plausibly be attributed to the inhibition of Aurora A, polo-like kinase (PLK1) but not Aurora B activity thereof [99]. Interestingly, BITC treatment for ovarian cancer cells led to the perturbation in the cell cycle progression, which increased the percentage of cells in the G2 and M phase; the mechanism of action behind the activity of BITC to cause G2/M cell cycle arrest was possibly due to the decreased Akt signaling [130].

#### 3.2.3. Impacts of PEITC on Modulation of Cell Cycle

As a matter of fact, heat shock proteins (HSPs) augment cell cycle progression through the increased activity of NF-κB; therefore, it has been envisaged that suppression of these HSPs may be a promising target for the development of cancer drugs. To this end, PEITC treatment significantly down-regulated different HSPs, including Hsp27, Hsp70, and Hsp90, which led to the cell cycle arrest at the G2/M phase, while PEITC decreased the percentage of cells in the G0/G1 and S phases, which ultimately led to reduced NF-κB activity in MDA-MB-231 and MCF-7 cells [100]. Furthermore, it has been reported that PEITC promoted the down-regulation of cyclin B1, CDK1, Cdc25C, and PLK-1 and increased the up-regulation of p53 and p21 in treated MDA-MB-231 and MCF-7 cells [101]. Research has also shown that PEITC treatment in MDA-MB-231 and MCF-7 cells resulted in the increment in the percentage of cells in G1 and G2/M, while a significant reduction in the percentage of cells in the S phase was observed in a dose- and time-dependent manner [132]. Another study showed that PEITC altered the expression of p57 in breast cancer cells, which is an inhibitor of cyclin D and cyclin E; thus, p57 contributes to cell cycle arrest at the G1-phase [134].

Collectively, the above collation of the literature distinctly highlights the importance of ITCs in the regulation of cell cycle arrest in female-associated cancers.

### 3.3. Impacts of Isothiocyanates on Apoptosis

It is widely known that apoptosis is an ordered and orchestrated cellular process. Morphological hallmarks of apoptosis include chromatin condensation and nuclear fragmentation, which are accompanied by rounding up of the cells, reduction in cellular volume (pyknosis), retraction of pseudopods, etc. At the later stages of apoptosis, some of the morphological features include membrane blebbing, ultrastructural modification of cytoplasmic organelles, and loss of membrane integrity [145]. It is widely known that conventional apoptosis mainly occurs either through an intrinsic mitochondrial pathway or through the extrinsic death receptor-mediated pathway. The mitochondrial death pathway is regulated by Bcl-2 family members and involves the down-regulation of Bcl-2 and Bcl-xL proteins as well as the up-regulation of pro-apoptotic factors that enhance the participation of several signaling pathways. The Bcl-2/Bax ratio is critical for cytochrome-c expression; a low Bcl-2/Bax ratio triggers the release of cytochrome-c from mitochondria, which activates caspase-3, which in turn activates the PARP cleavage that has been recognized as a hallmark of apoptosis. Poly (ADP-ribose) polymerase is a critical enzyme that is particularly involved in the DNA repair and modulation of chromatin structure. Several investigations have revealed that apoptosis may also be induced by oxidative stress [146,147,148,149]. It is reasonable to argue that an important goal of clinical oncology has been the exploration of therapeutic moieties promoting the effective elimination of cancer cells by apoptosis.

#### 3.3.1. Impacts of SFN on Apoptosis

To this end, interestingly, reports have shown that SFN treatment strongly induces apoptosis seemingly through elevating the expression of c-caspase-3, Bax, and c-PARP and down-regulating the expression of Bcl-2 and Bcl-xL in MCF-7 and MDA-MB-231 cells [73]. Several studies have indicated that apoptosis induction can occur through different mechanisms. DNA fragmentation was observed in SFN-exposed MCF-7 and MDA-MB-231 cells, which occurred in a dose-dependent manner. Particularly, in MDA-MB-231 cells, SFN-mediated apoptosis induction occurred in response to Fas ligand activity, leading to the activation of caspase-3 and caspase-8, and PARP inhibition. However, apoptosis in MDA-MB-468, MCF-7, and T47D cells was mediated through increment in the expression of cytosolic cytochrome-c, down-regulation of Bcl-2, and activation of caspase-3 and caspase-9, attributing to decreased expression of estrogen receptor-α, histone deacetylase, and EGFR [74]. Another study on the cytotoxic potential of SFN showed that apoptosis induction in SFN-treated MCF-7 cells occurs due to the suppression of dynamic instability and reduction in microtubule turnover [121]. Furthermore, SFN-treated HeLa cells showed increased apoptosis induction and reduced level of inflammation, which was seemingly linked with down-regulation of Bcl-2 and decreased signaling of IL-1β and COX-2, as also discussed above [84].

Furthermore, apoptosis induction in SFN-treated cervical cancer cells was found to be due to the decreased expression of Bcl-2, Bcl-xL, PARP, and β-catenin and enhanced the expression of Bax and caspase-3 [86]. Another study demonstrated that SFN treatment induced apoptosis in SKOV-3, C3, and T3 cells by increasing the expression of c-PARP in a concentration- and time-dependent manner; nevertheless, the authors have not studied other apoptosis-associated proteins such as Bax, Bcl-2, Bcl-xL, cytochrome-c, and intrinsic and extrinsic caspases [39]. Furthermore, SFN-induced apoptosis in MDAH-2774 cells, which was clearly evident from the flow cytometry data, wherein the number of annexin-V-positive and propidium iodide-positive cells increased with increment in the concentration of SFN, indicating accumulation of early and late apoptotic cells. Basically, SFN-induced apoptosis occurred in response to the elevated expression of Bax, caspase-9, and c-PARP, and reduced the expression of Bcl-2 [82]. SFN was very effective in inducing apoptosis in OVCAR-3 and A2780 cells seemingly by down-regulating Bcl-2 and Bcl-xL, along with the increment in the expression of Bax, caspase-3, and cytosolic cytochrome-c, and there was a significant inhibition of Akt and NF-κB proteins thereof [80].

#### 3.3.2. Impacts of BITC on Apoptosis

BITC-exposed MCF-7 and MDA-MB-231 cells showed considerable apoptotic induction, which was plausibly mediated by ROS generation through inhibition of complex III of the mitochondrial respiratory chain. These events eventually further potentiated Bax and caspase-3 overexpression and down-regulated catalase and superoxide dismutase. Moreover, BITC treatment induced apoptosis in MCF-7 and MDA-MB-231 cells seemingly by up-regulating Bax and Bak along with attenuation of Bcl-2 and Bcl-xL expression [88]. BITC treatment resulted in apoptosis induction in ovarian cancer cells as well. Interestingly, BITC inhibited Bcl-2 expression and up-regulated the expression of caspase-3, caspase-8, c-PARP, and Bax, seemingly via activation of JNK1/2/p38 and inhibition of ERK1/2 and Akt signaling in ovarian cancer cells [150]. Furthermore, BITC-exposed ovarian cancer cells showed significant apoptosis induction in a dose-dependent manner, which occurred in response to the DNA fragmentation and mitochondrial membrane depolarization, which was linked with the reduced expression of Akt protein [130].

#### 3.3.3. Impacts of PEITC on Apoptosis

PEITC exposure to MDA-MB-231 and MCF-7 cells significantly induced apoptosis, which resulted in decreased cell viability. The mechanistic insights into the mode of action of PEITC responsible for apoptosis induction in breast cancer cells were elucidated thereof. It was found that PEITC decreased the expression of Bcl-2 and triggered an increase in the expression of proapoptotic proteins such as Bax, caspase-3, caspase-9, and caspase-8, while at the same time, PEITC reduced the expression of mitochondrial cytochrome-c and promoted the expression of cytosolic cytochrome-c [101]. Furthermore, another study investigated the effects of PEITC on human breast cancer cells (MDA-MB-231 and MCF-7). Interestingly, it was found that PEITC readily induced apoptosis in MDA-MB-231 cells (as indicated by ready activation of caspases-9 and 3, and decreased expression of Bax); nevertheless, MCF-7 cells were relatively resistant to the apoptotic effects of PEITC. It was envisaged that the relative resistance of MCF-7 cells was seemingly associated with high basal expression of NRF2, a transcription factor that coordinates cellular protective responses to oxidants and electrophiles, and raised intracellular levels of GSH. Thus, differences in the basal expression of NRF2 and resultant changes in GSH levels seem to be an important determinant of sensitivity to PEITC-induced apoptosis [132]. Furthermore, another interesting study demonstrated that PEITC-induced apoptosis in breast cancer cells was independent of the p53 up-regulated modulator of apoptosis (PUMA), but the apoptosis induction occurred in response to the B-cell lymphoma 2 interacting mediator of cell death (Bim), Bax, Bak, and caspase-3 activation [104]. Moreover, DNA fragmentation, down-regulation of Bcl-2 and XIAP, Smac translocation, and release of cytochrome-c into the cytosol with concomitant PARP cleavage and decreased expression of procaspase-7 and procaspase-9 significantly contributed to PEITC-induced apoptosis in MCF-7 cells, indicating the involvement of the mitochondrial pathway [105].

PEITC exposure to cervical cancer cells resulted in the activation of death receptors 4 and 5 (DR4, DR5), which further led to apoptosis induction in these treated cells [108]. Moreover, the inhibitory effects of PEITC were due to the activation of caspase-3 and -8, which may be in response to the down-regulation of ERK and MEK pathways [108]. Accumulating evidence has highlighted the impact of PEITC on the up-regulation of apoptosis-associated proteins in HeLa cells.

PEITC-mediated cytotoxicity in CaSki and HeLa cells was triggered in response to the apoptosis induction through intracellular and mitochondrial ROS generation, which led to nuclear fragmentation and caspase-3 activation thereof [138]. Furthermore, PEITC-mediated cytotoxicity was due to a 4–6-, 2–5-, and 2–10-fold increase in apoptosis induction in OVCAR-3, SKOV-3, and TOV-21G cells, respectively, which is achieved through promoting the expression of c-caspase-3 and c-PARP [113]. PEITC treatment resulted in a reduction in phosphorylation of EGFR and Akt, leading to decreased EGFR and Akt activities in OVCAR-3, SKOV-3, and TOV-21G cells, which are positively correlated with the activation of caspase-3 and PARP cleavage [113]. Further investigations on the mode of action of PEITC revealed that PEITC targets the key signaling pathways of ovarian cancer because PEITC inhibited TGF-dependent activation of EGFR-Akt, and there was significant inhibition of Rictor, Raptor, mTORC1, and mTORC2 thereof [113]. The morphological analysis of PEITC-treated SKOV-3, OVCAR-3, and NUTU-19 cells revealed that PEITC induced morphological changes such as rounding, detachment, and floating cells which are the major hallmarks of apoptosis induction [87]. Furthermore, PEITC-mediated apoptosis induction was validated by increased activation of caspase-3, caspase-8, and caspase-9, and enhanced expression of Bax and c- PARP, while a significant reduction was noted in the expression of Bcl-2 in PEITC-exposed ovarian cancer cells [87]. Further investigations on the molecular mechanism of PEITC revealed that apoptosis induction in ovarian cancer cells occurred seemingly through increased phosphorylation of JNK1/2 and p38 and reduced phosphorylation of ERK1/2, Akt, and c-Myc, indicating PEITC targets JNK/p38 and ERK/Akt signaling pathways [87]. PEITC was cytotoxic to ovarian cancer cell lines, including OVCAR-3, SKOV-3, PA-1, CAOV3, and A2780, and PEITC promoted mitochondrial ROS generation, which thereby inhibited complex III of the electron transport chain leading to significant cell death in these ovarian cancer cells [114]. Another study demonstrated that PEITC induced ROS accumulation to activate apoptotic cascade via unfolded protein response (UPR) in ovarian cancer cells. Additionally, PEITC-mediated apoptosis occurred through up-regulating CHOP/GADD153 and Bip/GRP78 with concomitant activation of PERK and IRE1 in SKOV-3 and PERK and ATF-6 in PA-1 ovarian cancer cells. PEITC treatment caused excessive ROS production in order to inactivate redox-sensitive molecules, induce mitochondrial damage, and increase apoptosis induction which cumulatively showed the selective killing of ovarian cancer cells [151].

These scientific observations indicate that ITCs have significant potential to induce apoptosis in breast, cervical, and ovarian cancer attributed to the modulation of several molecular mediators involved in apoptotic responses.

### 3.4. Impacts of Isothiocyanates on Modulation of Autophagy

Autophagy is an evolutionarily conserved intracellular recycling system and cellular self-degradation process that maintains metabolism and homeostasis [152]. In cancer biology, autophagy plays dual roles in tumor promotion and suppression and contributes to cancer-cell development and proliferation [153,154]. It has been envisaged that autophagy is an attractive target for cancer therapeutics, and researchers have been exploiting the use of autophagy modulators as adjuvant therapy [155,156]. Interestingly, accumulating evidence has shown that isothiocyanates (SFN, BITC, and PEITC) trigger autophagic cell death seemingly through alteration of various signaling molecules, including mTOR, microtubule-associated protein 1 light chain 3 (LC3), beclin-1, p68, Akt, AMPK, PTEN, FOX1, etc. [91].

#### 3.4.1. Impacts of SFN on Modulation of Autophagy

An in vitro study showed that SFN induced considerable autophagy in MDA-MB-231, MCF-7, SKBR-3, and MDA-MB-468 cells. Interestingly, transmission electron microscopy (TEM) and fluorescence microscopic analysis showed vacuole formation and the presence of GFP-tagged LC3 protein in the vacuoles in the SFN-treated group. Furthermore, as we all know, mTOR is a negative regulator of autophagy, and SFN decreased the phosphorylation level of mTOR, leading to autophagy induction in triple-negative breast cancer cells (TNBCs) [70]. Another report has also envisaged that SFN-treated breast cancer cells showed autophagy induction, asserted by the presence of membranous vacuoles, autophagosomes and autolysosomes, and accumulated acidic vasicular organelles [73]. Furthermore, SFN exposure to breast cancer cells reduced ATP and lactate levels, which may be judged by AMPK activation, while SFN decreased the level of phospho-Akt without affecting glucose transporter 1 (GLUT1), hexokinase 2, lactate dehydrogenase A (LDHA), and pyruvate kinase isoform 2 (PKM2). This reduced energy stress in response to a decreased level of ATP, and AMPK activation promoted cytotoxic and cytostatic autophagy in the breast cancer cells [118]. Interestingly, SFN exposure to different breast cancer cells resulted in significant autophagy induction by increasing expression of LC3-I, LC3-II, and beclin-1, while a decline in the level of P62 was observed in treated cells [79]. Another preclinical study showed that SFN has a significant ability to induce autophagy in breast cancer cells (MDA-MB-231, BT549, and MDA-MB-468 cells) by targeting down-regulation of HDAC6 which increased translocation and acetylation of PTEN. Furthermore, mechanistic insights revealed SFN-mediated elevation in beclin 1 and LC3-II expression and down-regulated p62 expression levels in TNBC cells [79].

#### 3.4.2. Impacts of BITC on Modulation of Autophagy

TEM analysis revealed that BITC treatment resulted in the formation of double-membrane vacuoles resembling autophagosomes, acidic vasicular organelles, and cleavage of microtubule-associated protein 1 light chain into LC3-II in BITC-treated breast cancer cells [88]. Furthermore, breast cancer cells pre-treatment with autophagy inhibitors (3-methyl adenine and bafilomycin A1) resulted in partial but statistically significant attenuation of BITC-mediated growth inhibition [88]. Furthermore, FOX1-dependent autophagy was induced in human breast cancer through BITC administration [91]. Interestingly, autophagy induction and attenuated growth of MCF-7 cells were due to the cleavage of LC3, diminished activity of p68, and inhibition of mTOR activation [91].

#### 3.4.3. Impacts of PEITC on Modulation of Autophagy

Reports have shown that PEITC induces autophagy in various cancers [157,158]. It has been highlighted that PEITC induces autophagic cell death in prostate cancer cells, and it has been argued that PEITC-induced autophagy was plausibly regulated by Atg5 [158]. Furthermore, PEITC-mediated autophagy induction has also been reported in transgenic mice models of prostate cancer [159].

Albeit, there is information highlighting the impacts of PEITC on the modulation of autophagy in various cancers; nevertheless, as of now and to the best of our knowledge, reports highlighting the impacts of PEITC on the modulation of autophagy in female-specific cancer is rare.

Collectively, all these data envisage the importance of ITCs in the modulation of autophagic responses in female-specific cancer.

### 3.5. Impacts of Isothiocyanates on Cancer Stem Cells (CSCs)

Of note, CSCs are the subpopulations of tumor cells that actively participate in tumor initiation and progression. The key signaling pathway that acts as a major regulator for CSCs is the PI3K/Akt/mTOR signaling pathway [160]. It has been highlighted that various molecular mediators, including aldehyde dehydrogenase (ALDH), CD44, CD24, Nanog, Notch, c-Myc, Oct-4, Sox-2, KLF4, Slug, and Wnt/β-catenin signaling considerably contribute to CSC proliferation and metastasis. It has been envisaged that CSC formation occurs in response to chemotherapy, resulting in the failure of cancer therapy. Therefore, screening phytocompounds with the potential to limit the formation and spread of CSCs has been in demand. Thereby, various ITCs have been analyzed to inhibit the growth and proliferation of CSCs.

#### 3.5.1. Impacts of SFN on CSCs

Interestingly, it has been highlighted that SFN-treated MCF-7 and SUM159 cells showed reduced primary sphere formation, and there was a 65% and 80% reduction in the ALDH-positive cell population in SUM159 at 1 and 5 µmol/L concentration of SFN, respectively. Furthermore, SFN-treated animals also confirmed a decline in the number of ALDH-positive cells in breast tumors [75]. Mechanistic insights revealed that SFN-induced cytotoxic and antitumor effects were plausibly due to the down-regulation of the Wnt/β-catenin self-renewal pathway [75]. This down-regulation of Wnt/β-catenin in SFN-treated MCF-7 and SUM159 cells also led to a reduction in the cyclin D1 expression, which is a downstream protein of this signaling pathway, as highlighted in the above sections. SFN-treated TNBCs (MDA-MB-231-Luc-D3H1 and JygMC(A)GFP/Luc) showed proliferation inhibition, suppression of self-renewal of breast CSCs, and a reduced number and diameter of primary and tertiary tumor spheres, while SFN-treated CD49f+/CD24−/CD44+ breast CSCs showed a significant reduction in the number of secondary tumor spheres as well [161]. Interestingly, the study also highlighted that the tumor sphere forming-presumptive CSCs were more sensitive to SFN treatment than the unsorted bulk of TNBC cells. In comparison to the saline-treated animal group, SFN-pre-treated animals showed reduced gene expression of CSC markers, including Nanog, forkhead box D3 (FOXD3), Wnt3, ALDH1A1, and Notch4 during primary tumor growth. SFN (stabilized in alpha-cyclodextrin complex) treatment also inhibited breast CSC activity in primary and metastatic estrogen receptor-positive breast cancer patient-derived samples, seemingly by reducing mammosphere formation efficiency (MFE) and ALDH activity [162]. Importantly, the molecular mechanism responsible for the anti-bCSC action of SFN was revealed as a reduction in pSTAT3 levels and decrement in the number of ALDH-positive bCSCs and tumorigenicity [162].

#### 3.5.2. Impacts of BITC on CSCs

In vitro and in vivo studies have conferred the chemopreventive role of BITC, as it also inhibited the self-renewal of breast CSCs through reducing expression of Ron, N-cadherin, and vimentin along with E-cadherin overexpression [92]. An emerging paradigm suggests that BITC can also suppress CSC properties in different cancers by targeting various CSC-related marker proteins. Notably, B-lymphoma Moloney murine leukemia virus insertion region-1 (Bmi-1) and Notch4 serve as regulators of bCSC self-renewal and maintenance. BITC-treated MCF-7, SUM159, MDA-MB-231, MDA-MB-361, and MDA-MB-231 xenografts showed significant suppression of Bmi-1 [163]. Furthermore, BITC-mediated reduction in the ALDH1 activity was linked with the Bmi-1 expression in MDA-MB-231 and SUM159 cells, and over-expression of Bmi-1 in MCF-7 cells significantly attenuated BITC-dependent mammosphere formation [163]. BITC caused significant activation of Notch4, which indicated BITC possesses anti-breast CSC potential [163]. Another in vitro study demonstrated that BITC-exposed human breast cancer cells also increased the level of Notch1, Notch2, and Notch4, and the Notch activation was accompanied by induction of secretase complex component Nicastrin [94]. One of the interesting preclinical studies highlighted the role of BITC in targeting kruppel-like factor (KLF-4)-p21CIP1axis, which is generally implicated to be involved in the maintenance of bCSCs [164]. Moreover, BITC-treated MDA-MB-231, MCF-7, and SUM159 cells resulted in the induction of KLF-4 in a dose-dependent manner, and BITC also promoted KLF-4 mRNA expression [164]. KLF-4 knockdown augmented BITC-derived suppression of bCSCs, leading to a reduction in the ALDH-positive bCSC population both in MCF-7 and SUM159 cells. Furthermore, KLF-4 knockdown and BITC treatment are more effective in suppressing mammosphere formation when compared to either KLF-4 knockdown or BITC treatment alone, and KLF-4 knockdown resulted in the suppression of p211 in both MCF-7 and SUM159 cells, indicating bCSCs inhibition by BITC treatment is partially attenuated by the induction of KLF-4 and p21 [164].

#### 3.5.3. Impacts of PEITC on CSCs

PEITC treatment to chemo and radio-resistant breast cancer cells (MDA-MB-231/IR) resulted in intensive ROS generation, decreased metadherin (MTDH) expression, and reduced breast CSCs. Furthermore, the MTDH knockdown significantly promoted a reduction in ALDH activity and inhibition of CSC markers such as β-catenin, CD44, and Slug [103]. Furthermore, PEITC treatment resulted in the inhibition of the viability of ALDH-positive cells, decreased self-renewal ability, and inhibition of sphere formation efficiency in ovarian cancer (SKOV-3) and breast cancer cells (BT474, SKBR3, and HCC1954). Further investigations at the molecular level revealed that PEITC down-regulated phosphorylated HER2 monomer and reduced expression of Notch1 and Hes1 mRNA [112]. PEITC significantly attenuated the expression of ALDH1, resulting in the decreased cell viability of ALDH1-positive HeLa CSCs by increasing early apoptosis induction through the production of ROS and inhibition of Sp1 and promoter region of MDR1 (P-Gp) in PEITC-treated HeLa CSCs [110]. In comparison to MDA-MB-231 cells, radioresistant MDA-MB-231/IR cells showed higher expression of the CSC-associated marker proteins, and PEITC exposure to these cells suppressed cancer cell stemness through the down-regulation of CSC markers such as CD44, oct3/4, and Slug, and the down-regulation of metadherin protein at the post-transcriptional level [103].

In summation, it is reasonable to argue that the formation and spread of CSCs are considerably halted by ITCs, including SFN, BITC, and PEITC.

## 4. Combinatorial Studies on ITCs and Anticancer Drugs/Phytochemicals

Conventional chemotherapeutic regimes have been reported to pose many significant challenges, including adverse side reactions, systemic toxicity, low drug efficacy, and the development of drug resistance. It has been widely acknowledged that limited efficacy and development of resistance towards monotherapeutic drugs considerably reduce the opportunity to employ chemotherapeutic drugs for treating cancer patients. To this end, combination therapy, a treatment modality that combines two or more therapeutic agents, has shown a promising outcome; this intriguing approach potentially reduces the issue of drug resistance together with providing enhanced anti-cancer benefits in an additive or synergistic manner. In the following sections, we presented the prospective anticancer outcomes and the related mechanisms of the combinatorial therapeutic strategies of ITCs with the anticancer drug and/or with other phytochemicals. Interestingly, to this end, an in vitro study demonstrated that the combinatorial treatment of the chemotherapeutic drug lapatinib and ITCs significantly reduced SKBR-3 and BT-474 cell viability and induced apoptosis in a synergistic manner; mechanistically, it was highlighted that these enhanced anticancer effects were seemingly due to a considerable decrement in the phosphorylation status of HER2, Akt, and S6 [165]. Additionally, co-administration of lapatinib and ITCs greatly inhibited survival and migration of drug-sensitive and drug-resistant cell populations through decreased phosphorylation of Akt and VEGF in HER2-positive breast cancer cells [166].

### 4.1. Combinatorial Treatment of SFN and Anticancer Drugs/Phytochemicals

Accumulating evidence has highlighted the prospective potential of SFN in combinatorial therapeutic regimes with conventional chemotherapeutic drugs and/or with other phytochemicals with additive and/or synergistic potency. To this end, interestingly, it has been demonstrated that a combinatorial amalgam of SFN with withaferin A synergistically inhibited cell survival and impeded the cell cycle progression in MCF-7 and MDA-MB-231 cells [167,168]. Moreover, SFN potentiated the ability of paclitaxel and docetaxel to restrain and abolish bCSCs plausibly through suppression of NF-κB expression. Moreover, SFN and paclitaxel co-treatment considerably induced apoptosis seemingly by reducing Bcl-2 expression and enhancing expression of intrinsic and extrinsic proapoptotic proteins such as caspase-3, caspase-9, caspase-8, and cytosolic cytochrome-c, which was plausibly mediated through down-regulation of NF-κB and Akt, proclaiming increased paclitaxel sensitization of breast cancer cells [169]. Moreover, it has been highlighted that SFN and 5-FU co-administration significantly inhibited the survival of MDA-MB-231 cells in a synergistic manner through induction of cell death and senescence [170]. Interestingly, it has been shown that the combination of SFN and 5-FU has the ability to suppress the proliferation of the salivary gland adenoid cystic carcinoma high metastatic cell line and low metastatic cell line in a synergistic manner by reducing NF-κB p65 expression [171]. Furthermore, SFN and gemcitabine co-administration significantly reduced cell growth in a synergistic manner by inducing apoptosis in MCF-7 cells, and SFN also potentiated the sensitivity of gemcitabine towards breast cancer cells without having any significant adverse effects on normal cells [172]. Another study showed that co-administration of SFN and cisplatin considerably attenuated TNBC cell proliferation (MDA-MB-231 and MDA-MB-468) by inducing apoptosis and S phase cell cycle arrest; synergistically reduced TNBC cell migration and invasion by decreasing MMP2 and MMP9 expression; reversed epithelial–mesenchymal transition (EMT) process by suppressing sirtuins (SIRTs); and decreased cancer cell stemness by down-regulating N-cadherin, vimentin, Slug, and Snail; and altered chromatin modifications of E-cadherin promoter [173]. The combination of SFN and eugenol significantly reduced HeLa cell growth and survival, induced apoptosis by down-regulating Bcl-2, and suppressed the expression of inflammatory molecules such as COX-2 and IL-β. Gemcitabine used in conjunction with SFN and eugenol at higher doses leads to synergistic killing of HeLa cells by inducing apoptosis [174]. SFN and cisplatin cotreatment to OVCAR-3 and A2780 resulted in the reduction in cell proliferation, suppression of colony formation ability, and induction of apoptosis and cell cycle arrest in response to the decreased expression of Bcl-2, c-Myc, and cyclin D1 and up-regulation of p53 and c- caspase-3. SFN and cisplatin combination treatment showed increased cell viability inhibition and induced apoptosis in the ovarian cancer cell line (A2780 cells) in a synergistic manner, and SFN significantly enhanced cisplatin-mediated DNA damage in A2780 cells while SFN protected SKOV3 cells [175]. Moreover, the combinatorial treatment of three drugs, including SFN, EGCG, and cisplatin, has also been investigated. Interestingly, it was demonstrated that SFN and EGCG greatly potentiated cisplatin anticancer efficacy by increasing cell proliferation inhibition, apoptosis induction, and cell cycle arrest in cisplatin-sensitive as well as cisplatin-resistant cell lines. Upon examining the underlying mode of action, the combinatorial amalgam SFN, EGCG, and cisplatin were found to increase the expression of p21 at transcription and translation levels [176]. Likewise, Tollefsbol and group highlighted that tri-combinatorial treatment consisting of SFN, genistein, and sodium butyrate effectively inhibited breast cancer genesis, seemingly at least in part, through regulation of epigenetic modifications [177]. Furthermore, SFN effectively potentiated cisplatin efficacy towards the OVCAR3 cells, seemingly through blocking ovarian cancer cell proliferation and reversing c-Myb effects [178]. Additionally, SFN and cisplatin co-treatment considerably inhibited ovarian cancer cells by up-regulating miR-30a-3p, inducing DNA damage and reducing DNA repair and cisplatin accumulation, and reducing the ability to repair damaged DNA strands. As a result, co-treatment with SFN and cisplatin results in the reversal of cisplatin resistance [179]. The co-administration of SFN and cisplatin suppressed A2780 cell growth and caused DNA damage, and apoptosis induction occurred by the down-regulation of Nrf-2 expression and, as a result, cisplatin sensitization was found to be increased [175]. SFN and EGCG co-treatment inhibited 2780/CP20 (cisplatin-resistant) and A2780 (cisplatin-sensitive) ovarian cancer cells synergistically, which also potentiates cisplatin-based apoptosis induction and G2/M cell cycle arrest [180]. Moreover, SFN and EGCG co-administration suppressed the growth of ovarian cancer cells, caused G2/M cell cycle arrest, and induced apoptosis in SKOV3TR-ip2 cells (paclitaxel-resistant ovarian cancer cell line) by increasing the down-regulation of Bcl-2, hTERT, and PARP cleavage [176]. Additionally, SFN and tamoxifen co-treatment also decreased ALDH activity and MFE in breast cancer cell lines (MCF-7, T47D, and ZR-75-1) and primary breast cancer cells while tamoxifen either alone or in combination with SFN increased bCSC activity measured by ALDH activity and MFE, but SFN alone was effective in reducing ALDH activity and MFE in patient-derived xenografted animals [162]. Moreover, sequential treatment with SFN and 5-FU resulted in autophagic cell death, which was well evident from the autophagic vacuole accumulation. SFN and 5-FU combinations exerted their cytotoxic effects on breast cancer cell lines in a synergistic manner, and molecular insights revealed increased expression of LC3-I and LC3-II in the combination-treated group [170]. Furthermore, an interesting in vivo study reported that SFN increases doxorubicin efficacy seemingly by reducing the required doxorubicin dose, and also attenuated cardiotoxicity and regressed tumor growth when co-administered with doxorubicin in a rat orthotopic breast cancer model [181].

### 4.2. Combinatorial Treatment of BITC and Anticancer Drugs/Phytochemicals

Furthermore, SFN and BITC were also reported to enhance the sensitivity of cisplatin towards HeLa cells and induce apoptosis by PARP cleavage in HeLa, C33A, and MCF-7 cells [182]. Both ITCs sensitize cervical cancer cells to etoposide and adriamycin-mediated apoptosis by activating intrinsic and extrinsic caspases [183].

### 4.3. Combinatorial Treatment of PEITC and Anticancer Drugs/Phytochemicals

PEITC and paclitaxel co-administration synergistically inhibited cell growth and induced apoptosis and cell cycle arrest in MCF-7 and MDA-MB-231 cells, and mechanistically, it was reported that PEITC and paclitaxel combination increased acetylation of alpha-tubulin and down-regulated CDK1 and Bcl-2 along with the increase in Bax expression and PARP cleavage [184]. Another in vitro study on chemo- and radio-resistant breast cancer cell lines (MDA-MB-232/IR) found that PEITC inhibited breast cancer cell survival by triggering the production of ROS, and thus increased antioxidant gene expression [103]. PEITC was reported to enhance the anticancer effects of doxorubicin at lower doses in HER2-positive cancer cell lines (MDA-MB-231 and MDA-MB-231 high HER2), and upon exploring the molecular mechanism, the PEITC and doxorubicin combination was found to down-regulate HER2, EGFR, and phosphorylated STAT3 while up-regulating c-caspase-3 and PARP [185]. PEITC and doxorubicin co-administration exerted significant cytotoxic effects on MCF-7 breast cancer cells, attenuated cell migration, and induced apoptosis through increasing expression of c-caspase-3 and enhanced caspase-7 enzymatic activity. Moreover, combinatorial treatment of PEITC and paclitaxel inhibited breast cancer cells synergistically, induced apoptosis through enhanced Bcl-2 inhibition, PARP cleavage, and Bax overexpression, and also triggered G2/M cell cycle arrest by down-regulating expression of cyclin B1 and CDK1 in MCF-7 and MDA-MB-231 cell lines [184,186]. Furthermore, cervical cancer cells co-treated with PEITC and cisplatin showed inhibitory effects and induced apoptosis in HeLa and CaSki cells [138]. Another in vitro study showed that pre-treatment of HeLa cells with PEITC results in an increment in cisplatin-mediated cytotoxicity by inducing apoptotic cell death through PARP cleavage and activation of extracellular signal-related kinase in a synergistic manner. PEITC combined with cisplatin was reported to increase apoptosis by activating MEPK/JNK/ERK signaling in C33A and HeLa cells [182]. Furthermore, metformin and PEITC caused cellular and mitochondrial ROS generation in ovarian cancer cells that leads to apoptosis induction and inhibition of ovarian cancer cell growth in a synergistic manner. Additionally, both compounds together exerted synergistic anti-ovarian cancer effects on a cisplatin-resistant cell line (A2780cis) [114]. PEITC was reported to potentiate the cytotoxic effects of the PARP inhibitor BMN 673 against ovarian cancer cells through ROS generation and accumulation, elevated DNA damage, enhanced apoptosis, and G2/M cell cycle arrest. Moreover, the PEITC and BMN673 combination also attenuated the growth of ovarian tumor spheroid and patient-derived organoid models of high-grade serous ovarian cancer and cervical cancer [187]. Additionally, the PEITC and doxorubicin combination reduced tumor volume and tumor weight and increased the survival rate of tumor-bearing mice. The mechanistic insights revealed that PEITC and doxorubicin co-administration decreased the p-Akt/Akt ratio and suppressed NF-κB p65 DNA binding activity in the MCF-7 cell line [188].

## 5. Isothiocyanates and Their Anticancer Potential in Animal Model

As a matter of fact, in the progression from bench to bedside for anticancer therapeutic drugs, it is widely acknowledged that prudent use of preclinical screening models seemingly provides a great deal of information on the preliminary efficacy, toxicity, pharmacokinetic, and safety information regarding the drug under investigation. Needless to say, all this information helps research fraternities decide whether or not to support their further clinical trial evaluations. In the context of this review, the preclinical studies highlighting the efficacy of ITCs in various animal models have been summarized in the following sections.

### 5.1. SFN and Their Anticancer Potential in Animal Model

Various studies have highlighted the intriguing anticancer potential of SFN in animal models of breast, cervical, and ovarian cancer. To collate a few, interestingly, SFN exerted intriguing anticancer effects in nude mice xenotransplanted with MDA-MB-468 cells. In comparison to the untreated control group of animals, SFN treatment significantly inhibited primary tumor growth seemingly through the reduction in the tumor volume and tumor size in treated animal groups, attributed to a decrement in proliferation ratio and elevation in the apoptotic ratio of primary tumor cells. Additionally, SFN treatment considerably reduced lymph node metastasis in KPL-1 breast cancer cell xenografts in female athymic mice [122]. Intriguingly, SFN significantly reduced tumor size and tumor weight by 60 and 70%, respectively, in TNBCs. Interestingly but not surprisingly, hematoxylin and eosin staining of the tissues showed that SFN caused no or minimal adverse side effects upon oral administration [122]. Furthermore, SFN efficacy in athymic nude mice model injected with A2780 cells was also studied. The results showed that SFN treatment considerably suppressed tumor growth, seemingly through the reduction in tumor size and tumor weight in the treated animal group. Moreover, immunohistochemical staining confirmed that SFN caused a reduction in the expression of KI67 and Her2. Thereafter, Western blot analysis of the tissue samples showed a decrement in the expression levels of c-Myc, Bcl-2, cyclin-D1, and Her2, and an increment in the expression of Bax, p53, and p27 thereof [80]. Likewise, SFN efficacy in NOD/SCID mice (xenograft model) inoculated with SUM159 cells was investigated. SFN treatment (50 mg/kg) suppressed breast tumor growth by 50% as compared to the untreated control group; interestingly, no significant toxic effects were observed in SFN-treated animal groups [75]. Furthermore, SFN containing broccoli sprouts and genistein combinatorial treatment significantly reduced mammary tumor incidence, tumor volume, and delayed tumor latency in a transgenic breast cancer mouse model [189].

### 5.2. BITC and Their Anticancer Potential in Animal Model

BITC treatment exerted inhibitory effects on proliferation, migration, and angiogenesis by down-regulating VEGF receptor 2 in MDA-MB-231 xenografted cells, proving BITC efficacy against breast cancer [93]. BITC-treated breast cancer cell lines (MDA-MB-231, MCF-7, MDA-MB-468, BT-474, and BRI-JM04) and MDA-MB-231 xenograft model mice showed induction of autophagic cell death, which is evident from features of autophagy such as the appearance of double-membrane vacuoles, acidic vesicular organelles, suppression of p62 expression and cleavage of microtubule-associated protein 1 light chain 3 (LC3). Furthermore, BITC-mediated autophagy was due to the increased expression and acetylation of FoxO1 and reduced phosphorylation of mTOR, P70s6k, and 4E-BP1 in xenograft mice [91]. BITC suppressed tumor growth by reducing cell proliferation, migration, and neovascularization in MDA-MB-231 breast cancer xenografts. Interestingly, BITC-administered tissues showed down-regulated expression of Ki-67, VEGF receptor 2 proteins, and suppressed VEGF secretion [93]. BITC administration at a dose of 1 and 3 mmol/kg resulted in the prevention of mammary cancer in MMTV-neu mice by suppressing the incidence and/or burden of mammary hyperplasia and carcinoma. Interestingly, BITC administration-mediated prevention of breast carcinogenesis was further correlated with cell proliferation inhibition and apoptosis induction [190]. Another in vivo study showed that BITC treatment resulted in tumor suppression in breast tissues, which is clearly due to the decreased levels of Drp1, Fis1, and Mfn1 in BITC-treated groups [191]. 4T1 mammary carcinoma cells were injected into the inguinal mammary fat pad of syngeneic female BALB/c mice, and after a day, the animal groups were administered with BITC (0, 5, and 10 mg/kg/body weight/day) for up to 28 days, which then resulted in the reduction in tumor volume and tumor weight. BITC treatment significantly down-regulated proliferating cell nuclear antigen (PCNA), Bcl-2, VEGF, and CD31, while decreasing enhanced expression of Bax, caspases, and c- PARP in BITC-treated animal groups [192]. Another in vivo study demonstrated suppression of murine mammary carcinoma cell growth and metastasis, and apoptosis induction by BITC, which is then correlated with the altered Wnt/β-catenin signaling [193]. Furthermore, BITC treatment markedly inhibited high-fat diet-stimulated mammary tumor progression and metastasis in obesity-resistant BALB/c mice. BITC reduced solid tumor growth and the number of tumor nodules in the lung and liver in the control diet group of mice as compared to the BITC-untreated high fat diet feeding group of mice [194].

### 5.3. PEITC and Their Anticancer Potential in Animal Model

An interesting report by Srivastava and group has highlighted that PEITC exerted intriguing anticancer effects in immunocompromised NOD-SCID IL2Rγ^−/−^ (SCID/NSG) mice bearing MDA-MB-231 xenografts [107]. Their study explicitly highlighted that oral administration of PEITC considerably attenuated tumor growth by over 76%. Interestingly, this marked tumor-inhibitory phenotype was associated with a significant reduction in the levels of Myeloid-derived suppressor cells (MDSCs) bearing the surface markers CD33, CD34, and CD11b in PEITC-treated animals. To this end, the group envisaged that the overall tumor growth suppression by PEITC was considerably associated with the attenuation of MDSCs. Furthermore, PEITC treatment suppressed migration and invasion of breast cancer cells to the brain tissue in a murine model of breast cancer metastasis, which was 50% less than in the untreated animal group. Additionally, PEITC treatment also prolonged the survival of tumor-bearing mice by up to 20.5% [195]. Furthermore, PEITC and trastuzumab were tested for their antitumor potential in transgenic mice injected with MI6 tumor cells, and the results showed that PEITC inhibited the growth of MI6 nodules and significantly reduced spontaneous tumor development in d16HER2 transgenic mice when combined with trastuzumab [112]. Likewise, the antitumor efficacy of PEITC was further investigated in immunocompromised NOD-SCID IL2Rγ^−/−^ host mice bearing the MDA-MB-231 xenograft model of breast cancer. The results showed that PEITC treatment significantly reduced breast tumor growth by 76%, which was markedly supported by a reduction in the levels of myeloid-derived suppressor cells bearing surface markers such as CD11b, CD33, and CD34 [107].

Furthermore, PEITC was demonstrated to induce anti-metastatic effects in the intraperitoneal xenograft model of ovarian cancer; intriguingly, PEITC inhibited cell migration and invasion, which was explicitly evident from the decreased number of metastases in the stomach, liver, spleen, small intestine, and diaphragm of the PEITC-treated animal group, whereas the control animal group of ovarian cancer showed an elevated number of metastases in those organs. Additionally, the underlying mechanism of action of PEITC responsible for its antitumor properties seemingly involves the down-regulation of mTOR and chromosome region maintenance 1 protein (CRM1) proteins [111]. Preclinical studies on an ovarian cancer mouse model clearly showed that an oral dose of PEITC significantly suppressed ovarian cancer tumor growth seemingly by targeting inhibition of the EGFR-Akt pathway [113].

## 6. Isothiocyanates in Clinical Trials

Existing data from in vitro and in vivo studies plausibly warrant the intriguing anticancer potential of ITCs. Therefore, selective ITCs have been evaluated for their antitumor efficacy in human clinical trials. This perspective section summarizes ITCs and their clinical trials against cancer (www.clinicaltrials.gov, accessed on 9 April 2023). Summary of some of the clinical trials of isothiocyanates is presented in Supplementary Information (Table S1).

As a matter of fact, one of the interesting studies evaluated the anticancer effects of ITCs from broccoli sprouts in postmenopausal breast cancer patients. Basically, thirty patients were randomly assigned to receive either ITC-rich broccoli sprout extract (BSE) or a placebo for two weeks. Although biomarker changes at the breast cancer tissue level were not statistically significant, trends of increase in cleaved caspase 3 and tumor-infiltrating lymphocytes (TILs) and decrease in Ki-67 and nuclear to cytoplasm ratio of estrogen receptor (ER)-*α* were observed in the BSE arm, supporting ITC-induced activation of apoptosis and immune function but inhibition of ER-*α* signaling and cellular proliferation. These tissue-level effects observed in the BSE arm were confirmed by global evaluation of urinary proteomic profiles between pre- and post-intervention. Interestingly, a total of 116 urinary proteins were altered specifically by the BSE intervention involving 55 enriched signaling pathways, of which multiple pathways were known related to ITC functions. The changes in specific biomarkers at the breast cancer tissue level and the changes in global proteomic profiles at the individual level are highly concordant to support anticancer effects of ITCs on breast cancer. Of note, these findings are consistent with the anticancer mechanisms of ITCs identified in in vitro and in vivo studies. The study supports the potential beneficial roles of ITC-containing cruciferous vegetables in breast cancer prognosis [196]. Further, interestingly but not surprisingly, a phase I trial investigated the effect of healthy eating program (increasing the intake of cruciferous vegetables) in improving outcomes in patients with bladder cancer. The measurable outcome of this study was to attain desirable ITC levels; nevertheless, so far, no finding is reported as still the trial is ongoing [197].

Further, in another study of the same series, the focus was to understand the effects of PEITC on oral cells with mutant p53 in heavy smokers who are at higher risk of oral cancer. This study could provide a solid proof for the potential of ITCs for chemo-preventive and/or oncologic treatment of individuals with oral cancer. Unfortunately, the results of the trials were not released officially [198]. 

Meanwhile, another study focused to understand the anticancer effects of SFN against prostate cancer. Basically, the study investigated the mechanisms by which SFN altered the gene expression via epigenetic modifications and inhibition of histone deacetylase (HDAC) in human colorectal and prostate cancer cells. On the whole, the trial aimed to investigate the effects of short-term supplementation of SFN-rich BSE on benign epithelial tissue in men at risk for prostate cancer [199]. Mainly the study involved 98 subjects aged 50–78 years, with 50 in the BSE group and 48 in the placebo group. The compliance rate was 84% for the BSE group and 85% for the placebo group, with no statistically significant difference in compliance between the groups. The BSE supplement was found to have significant interactions in gene expression related to prostate cancer development, but did not significantly alter expected prostate cancer biomarkers. The study showed downregulation of two genes previously implicated in prostate cancer development, AMACR and ARLNC1, and higher levels of urine and plasma SFN ITCs and individual SFN metabolites in the treatment group. However, no significant difference in HDAC activity or prostate tissue biomarkers was reported.

Another study highlighted the potential chemo-preventive effects of consuming mustard (contains the active compound allyl ITC), which is widely consumed in Europe and the United States in salad dressings, sauces, or relishes. [200]. The findings suggest that short-term consumption of even low amounts of hot mustard, may be sufficient to protect cells from genotoxins. This is important since ITC from raw plants containing the prodrugs have reduced oral bioavailability rates [200]. Overall, the study, being the first trial examining the chemo-preventive effects of mustard consumption and highlighting the importance of verifying in vitro data with human intervention trials. The findings suggest that further research is needed to determine the potential impact of mustard consumption on lipid metabolism and to investigate the underlying mechanisms of the observed chemo-preventive effects [200].

On another note, two clinical trials are being conducted to investigate the potential use of PEITC as a chemo-preventive agent for lung cancer in smokers. Basically, the study aimed to provide insight into the potential use of PEITC as a chemo-preventive agent for lung cancer in smokers [201]. The first trial is a Phase 2 clinical trial that was divided into short-term and long-term trials. The short-term trial was conducted over a month, and participants were randomized to receive either oral PEITC or a placebo. The long-term trial was conducted over 12 months, where participants received oral PEITC or a placebo twice daily. Patients were stratified according to GST genotypes, and only those participants who met certain criteria may proceed to the long-term trial. Urine samples were examined for various biomarkers using LC-ESI-MS/MS, and tissue samples were examined for Ki-67, TUNEL, and caspase-3 expression using IHC [201]. The primary outcome measures included urinary levels of biomarkers of NNK metabolism and the effects of GSTM1 genotype on PEITC’s impact on urinary biomarkers of NNK metabolism. The secondary outcome measures included the effects of GSTT1 genotype and the combined effects of GSTM1 and GSTT1 genotype on PEITC’s impact on urinary biomarkers of NNK metabolism. The researchers Yuan et al. reported that PEITC has a 7.7% inhibitory effect on NNK metabolism in smokers, with stronger effects observed in subjects with the null genotype of both GSTM1 and GSTT1, women, subjects 40 years or older, and those with higher total 3′-hydroxycotinine: total cotinine ratio, a phenotypic measure of CYP2A6. They also reported a statistically significant effect of PEITC on glucuronidation of 3′-hydroxycotinine (a 22.9% increase) and borderline significantly increased urinary total nicotine (by 8.9%) and total nicotine equivalents (by 6.0%). The second trial which no official results reported was a Phase 1 clinical trial that aimed to determine the maximum tolerated dose of oral PEITC in smokers and to measure the steady-state pharmacokinetics of the substance required to maintain a steady state during exposure to NNK. Patients received escalating doses of PEITC orally four times a day for 30 days. Cohorts of 3–6 patients were used, and the maximum tolerated dose was determined when 2 of 6 patients experience dose-limiting toxicities [202]. A total of 15–27 patients were enrolled in the study. Asymptomatic smokers with urinary cotinine levels greater than 100 ng/mL were included in the study, and they must adhere to certain dietary restrictions limiting the intake of cruciferous vegetables while on the study. Patients must also meet certain eligibility criteria related to age, performance status, hematopoietic, hepatic, renal, and pulmonary function [202].

It is interesting to state that the clinical trials evaluating the antitumor efficacy of various ITCs have been conducted, including a phase II study. The study’s findings highlight the importance of incorporating cruciferous vegetables in the diet as a potential preventive measure. Nevertheless, more clinical studies are needed to determine the efficacy and safety of ITCs in cancer prevention and treatment.

## 7. Conclusions

Accumulating evidence highlights that plant-based bioactive molecules embody an intriguing potential to fill the void of limited chemotherapeutic options. Interestingly, several in vitro and in vivo studies have demonstrated the chemopreventive/chemotherapeutic role of many phytochemicals in female-specific cancers. In particular, ITCs have gained tremendous attention because of their tendency to target various cellular processes such as cancer cell growth, proliferation, migration, invasion, angiogenesis, and so on. Indeed, many studies strongly suggest the cytotoxic, anti-proliferative, anti-metastatic, antiangiogenic, anti-inflammatory, and proapoptotic effects of ITCs against female-specific cancers, as detailed above. Nevertheless, more and more being gleaned about their molecular intricacies would be highly instrumental in ITC-based pharmacological intervention providing an alternative and cost-effective option for cancer therapeutics. Nonetheless, these phytochemicals, in general, and ITCs, in particular, to be translated from bench to bedside require many daunting challenges to be overcome. Accordingly, more concerted efforts need to be diverted towards repurposing these plant-based phytochemicals, such as chemical modification of the pharmacophores, development of target-based delivery strategies including nano-formulations, their uses in adjuvant settings, and so on, in order to fully harness their true potential.

## Figures and Tables

**Table 1:** Representative table highlighting various Isothiocyanates, including Sulforaphane (SFN), Benzyl isothiocyanate (BITC), and Phenethyl isothiocyanate (PEITC), along with their structure and plant sources. Structures are drawn with ChemDraw Online Software.

Isothiocyanates	Structure	Sources of the Compound
Sulforaphane (SFN)	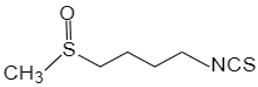	Broccoli, watercress, cauliflower, cabbage, kale, brussels sprouts, broccoli sprouts, etc.
Benzyl isothiocyanate (BITC)	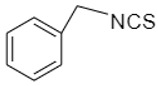	Broccoli, watercress, cauliflower, cabbage, pink mustard, papaya seeds, pilu tree, etc.
Phenethyl isothiocyanate (PEITC)	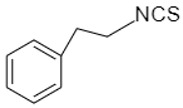	Broccoli, watercress, radish, turnip, cauliflower, cabbage, etc.

**Table 2:** Representative table highlighting the molecular intricacies of various isothiocyanates against female-specific cancers.

Phytochemicals	Cancer Model Studied	Molecular Mechanism	Major Findings	References
Sulforaphane (SFN)	MCF-7, MDA-MB-231, SKBR-3 and MDA MB 468	Decreased phosphorylation of Akt and S6K1	Growth Inhibition	[70]
	MCF-7, MDA-MB-231	Suppression of hTERT and down-regulation of DNMT1 and DNMT3a	Proliferation Inhibition	[71]
	MCF-7	Inhibition of estrogen receptor alpha protein and inhibition of progesterone receptor	Proliferation Inhibition	[72]
	MCF-7, MDA-MB-231	Activation of Caspase-3, Bax, and p21, and down-regulation of Bcl-2, cyclin A, cyclin B1, and Cdc2	Apoptosis, Cell Cycle Arrest Autophagy Inhibition	[73]
	MDA-MB-468, MCF-7 and T47D	Down-regulation of EGFR, HDAC, Bcl-2, caspase-3, and caspase-9	Apoptosis	[74]
	MCF-7 and SUM159	Down-regulation of Wnt/β-catenin	Apoptosis Induction, Cell Viability Inhibition, CSCs Inhibition	[75]
	MDA-MB-231, MCF-7 and SKBR-3	Down-regulation of Akt and DNMT and activation of p21 and p27	Apoptosis, Cell Cycle Arrest	[76]
	MDA-MB-468, MCF-7 and BT-474	Inhibition of HDAC5 and LSD1 Axis	Growth Inhibition	[77]
	MCF-7	Suppression of NF-κB signaling pathway and inhibition of TPA-induced MMP-9 expression	Proliferation Inhibition	[78]
	MDA-MB-231, BT549 and MDA-MB-468	Down-regulation of HDAC6 and increased membrane translocation and acetylation modification of PTEN	Growth Inhibition and Modulation of Autophagy	[79]
	OVCAR3 and A2780	Reduced Akt and NF-κB signaling; down-regulation of Bcl-2, Bcl-xL, c-Myc, and cyclin D1	Apoptosis and Cell Cycle Arrest	[80]
	SKOV3	Reduced Akt and PI3K signaling to control the expression of cyclin D1 and CDK4/6	Anti-Proliferative and Cell Cycle Arrest	[39]
	PA-1	Reduced cyclin B1 and Cdc2 expression	Cell Cycle Arrest	[81]
	MDAH2774 and SKOV3	Enhanced activity of Rb, c-PARP, and increased Bak/Bcl-2 ratio	Anti-Proliferative, Apoptosis Induction, and Cell Cycle Arrest	[82]
	A2780 and A2780/CP	Reduced AP1 and HIF-1 expression	Anti-proliferative and Anti-Metastatic	[83]
	HeLa, Cx, and CxWJ	Down-regulated cyclin B1 and up-regulated GADD45β	Growth Inhibition and Cell Cycle Arrest	[84]
	HeLa	Down-regulated Bcl-2, IL-1β, and COX-2	Growth Inhibition, Apoptosis, and Anti-Inflammatory	[84]
	HeLa	Targeted HDAC and DNMT3b	Apoptosis and Cell Cycle Arrest	[85]
	HeLa	Promoted Bax, caspase-3, and PARP cleavage while inhibited Bcl-2 and Bcl-xL	Apoptosis	[86]
Benzyl isothiocyanate (BITC)	MCF-7 and MDA-MB-231	Targeted JNK and p38 MAPK to generate excessive ROS activating Bax and Caspase-3	Apoptosis and Growth Inhibition	[87]
	MCF-7 and MDA-MB-231	Down-regulation of Bcl-xL, Bcl-2, cyclin B1, and CDK1, and up-regulation of Bax and Bak expression	Cell Growth Inhibition, Apoptosis, and Cell Cycle Arrest	[88]
	MDA-MB-231 and in vivo	Down-regulation of vimentin, fibronectin, Snail, and c-Met	Inhibition of EMT	[89]
	SUM159, MDA-MB-231 and in vivo	Up-regulation of E-cadherin and repressed uPA along with vimentin expression inhibition	Anti-Metastatic and Inhibition of EMT	[90]
	MCF-7	Promoted LC3 cleavage and reduced mTOR and p68 expression	Autophagy Cell Death and Growth Inhibition	[91]
	MCF-7 and in vivo	Inhibited expression of vimentin and N-cadherin and overexpressed E-cadherin	Inhibition of CSCs	[92]
	MDA-MB-231 and in vivo	Down-regulation of VEGF receptor-2	Anti-metastatic and Anti-Angiogenic	[93]
	MCF-7, SUM159 and MDA-MB-231	Activation of Notch2 signaling	Anti-Metastatic and Anti-Proliferative	[94]
	MCF7, MDA-MB-231	Down-regulation of Bcl-xL and Bcl-2 and up-regulation of PUMA	Apoptosis and Growth Inhibition	[95]
	MDA-MB-231 and MCF-7	Down-regulation of FOXH1 and Wnt/β-catenin	Growth and Invasion Inhibition	[96]
	MCF-7	Activation of p53-LKB1 and p73-LKB1 axes	Growth Inhibition	[97]
	HeLa	Reduced ATP levels and cause DNA fragmentation	Growth Inhibition and Apoptosis	[98]
	HeLa	Inhibition of Aurora A and PLK1 expression	Cell Cycle Arrest	[99]
Phenethyl Isothiocyanate (PEITC)	MCF-7 and MDA-MB-231	Reduced expression of HIF-1α, VEGF, and MMP2/9	Growth Inhibition	[100]
	MCF-7 and MDA-MB-231	Inhibition of HSPs (particularly HSP 90) and HSF1 Reduced expression of anti-apoptotic Bcl-2 protein, CDK1, and Cdc25C and increased expression of caspases, Bax, p21, and p53	Apoptosis and Cell Cycle Arrest	[101]
	MCF-7, H3396, SKBR-3 and MDA-MB-231	Down-regulation of estrogen receptor-α36 and abrogation of MAPK/ERK1/2 signaling	Growth Suppression	[102]
	MDA-MB-231/IR	Down-regulation of Metadherin, CD44, Slug, and β-catenin	Inhibition of CSCs	[103]
	BRI-JM04 MCF-7, and MDA-MB-231	Up-regulation of Bak, PUMA, and Bim (long and short forms of Bim), increased S65 phosphorylation of BimEL (extra-long form), and down-regulation of Bcl-xL and Bcl-2	Apoptosis and Growth Inhibition	[104]
	MCF-7	Down-regulation of Bcl-2/XIAP, up-regulation of procaspase-7/-9, PARP cleavage	Apoptosis and Cell Survival Inhibition	[105]
	MCF-7 and MDA-MB-231	Epigenetic reactivation of CDH1, down-regulation of Wnt/β-catenin signaling, and Inhibition of HDAC and DNMT expression	Inhibition of CSCs	[106]
	In vivo (Mice having MDA-MB-231 xenografts on MDSCs)	Inhibition of myeloid-derived suppressor cells (MDSCs)	Anti-Tumor Activity	[107]
	HEp-2 and KB	Activation of DR4 and DR5 through inactivation of ERK and MEK	Growth Inhibition and Apoptosis	[108]
	HeLa	Activation of TGF-β/Smad2 signaling pathway, reduced the expression of CDK1, MMP-2/9, CD44, and ICAM-1, increased the production of TGF-β, IL-6, and IL-8, and increased the phosphorylation of Smad2	Anti-Metastasis and Cell Cycle Arrest	[109]
	HeLa	Inhibition of Sp1 transcription factor and downstream multidrug resistance protein (P-glycoprotein)	Inhibition of CSCs	[110]
	SKOV-3, HO8910 and in vivo	Suppression of MMPs and mTOR-STAT3 signaling	Anti-proliferative and Anti-Metastatic	[111]
	HER2-positive BT474, SKBR3, HCC1954, SKOV3 and in vivo	Reduction in Notch1 and HER2 expression	Inhibition of CSCs, and Growth Inhibition	[112]
	SKOV-3, OVCAR-3, TOV-21G and in vivo	Inhibition of EGFR and Akt	Growth Inhibition and Apoptosis	[113]
	SKOV-3, OVCAR-3 and CAOV-3	Induced excessive ROS production	Apoptosis	[114]
	SKOV-3 and PA-1	Up-regulation of the key regulator of UPR-mediated apoptosis, CHOP/GADD153, and endoplasmic reticulum resident chaperone BiP/GRP78 along with activation of two major sensors of the UPR (PERK and ATF-6 in PA-1; PERK and IRE1α in SKOV-3)	Apoptosis	[115]

Abbreviations: AP1—activating protein-1, CDK4/6—cyclin dependent kinase 4/6, COX-2—cyclooxygenase-2, c-PARP—cleaved poly (ADP-ribose) polymerase, CSCs—cancer stem cells, DNMT—DNA methyltransferase, DR4/5—death receptor 4/5, EGFR—epidermal growth factor receptor, EMT—epithelial–mesenchymal transition, ERK1/2—extracellular signal-regulated kinase ½, GADD45β—growth arrest and DNA-damage-inducible gene β, HDAC—histone deacetylase, HER2—human epidermal growth factor receptor 2, HIF-1—hypoxia inducible factor-1, hTERT—human telomerase reverse transcriptase, HSPs—heat shock proteins, HSF1—heat shock factor 1, JNK—Jun N-terminal Kinase, LC3—microtubule-associated protein 1A/1B-light chain 3, MAPK—mitogen-activated protein kinase, MEK—mitogen-activated protein kinase kinase, MMP2/9—matrix metalloproteinase 2/9, NF-κB—nuclear factor kappa B, PTEN—phosphatase and tensin homolog, PLK1—polo-like kinase 1, PUMA—p53 up-regulated modulator of apoptosis, ROS—reactive oxygen species, TGF-β—transforming growth factor, uPA—urokinase plasminogen activator, and VEGF—vascular endothelial growth factor.

## Supplementary Materials

The following supporting information can be downloaded at: https://www.mdpi.com/article/10.3390/cancers15082390/s1, Table S1: Summary of Some of the Clinical Trials of Isothiocyanates.

## Abbreviations

AP1	activating protein-1
AMPK	AMP-activated protein kinase
Bcl-2	B-cell leukemia/lymphoma 2
Bcl-xL	B-cell lymphoma-extra large
BITC	benzyl isothiocyanate
CDH1	cadherin 1
CDK	cyclin-dependent kinase
DAPK1	death-associated protein kinase 1
ECM	extracellular matrix
EGCG	epigallocatechin gallate
ERK	extracellular signal-regulated kinase
FOX1	forkhead box O1
GLUT-1	glucose transporter-1
GRP78	glucose-regulating protein 78
GSK3-β	glycogen synthase kinase 3-β
GSTP1	glutathione S-transferase Pi
HIF-1	hypoxia-inducible factor-1
HPV	human papilloma virus
ICAM1	intercellular adhesion molecule
INK4	inhibitors of CDK4
JNK	Jun N-terminal Kinase
KLF4	kruppel-like factor 4
MAPK	mitogen-activated protein kinase
NF-κB	nuclear factor kappa B
NOD/SCID	nonobese diabetic/severe combined immunodeficiency
Nrf-2	nuclear factor erythroid 2–related factor 2
Oct-4	octamer-binding transcription factor 4
PEITC	phenethyl isothiocyanate
PERK	protein kinase R (PKR)-like endoplasmic reticulum kinase
PI3K	phosphatidyl inositol 3 kinase
RARβ	retinoic acid receptor β
ROS	reactive oxygen species
SFN	sulforaphane
SMAD	suppressor of mothers against decapentaplegic
STAT	signal transducer and activator of transcription
TGF-β	transforming growth factor
TNBCs	triple-negative breast cancer cells
VEGF	vascular endothelial growth factor
XIAP	X-linked inhibitor of apoptosis protein
