# Supplementary material for: Reviewing the Prospective Pharmacological Potential of Isothiocyanates in Fight against Female-Specific Cancers
Source: Cancers (Basel). 2023 Apr 20;15(8):2390. doi: 10.3390/cancers15082390 (PMC10137050; doi:10.3390/cancers15082390)
Supplement: Supplementary file 1 [file cancers-15-02390-s001.zip › cancers-2085000-supplementary.pdf]

Supplementary Information

Table S1. Summary of Some of the Clinical Trials of Isothiocyanates (Information extracted from clinicaltrials.gov).

Title of the clinical studies	Behavioural Dietary Intervention for the Improvement of Bladder Cancer Survivorship	Study of the Effects of PEITC on Oral Cells with Mutant p53	Chemoprevention of Prostate Cancer, Histone Deacetylase (HDAC) Inhibition and DNA Methylation	PEITC in Preventing Lung Cancer in Smokers	PEITC in Preventing Lung Cancer in People Who Smoke
Status	Recruiting	Completed	Completed	Completed	Completed
Conditions	Stage 0a Bladder Cancer AJCC v8	Oral Cancer	Prostate Cancer	Lung Cancer;<br>Tobacco Use Disorder	Lung Cancer
Interventions/ Treatment	Dietary Intervention	Dietary Supplement (Watercress Juice)	SFN-rich broccoli sprout extract (BSE) capsules	PEITC	PEITC
Outcome Measures	Changes in urinary ITCs levels	Evidence of reduced number of oral cells with mutant p53 following administration of PEITC derived from watercress	Changes in total urine SFN metabolites levels; changes of total Plasma SFN metabolites levels; changes in percentage of Ki67 Positive Cells post-randomization; changes in expression of HDAC6	Urinary levels of biomarkers of 4-(methylnitrosamino)-1-(3-pyridyl)-1-butanone (NNK) metabolism; effects of GSTM1 Genotype on PEITC-NNK association and on the metabolism and excretion of PEITC;<br>effects of GSTT1 Genotype on PEITC-NNK association and on the metabolism and excretion of PEITC;<br>combined effects of GSTM1 and GSTT1 Genotype on PEITC-NNK association and on the metabolism and excretion of PEITC; urinary Levels of [Pyridine-D4] Hydroxy Acid:Total [Pyridine-D4] NNAL ratio by GSTM1 and GSTT1 Genotype	No data
Sponsors/ Collaborators	Roswell Park Cancer Institute	Georgetown University	Portland VA Medical Center; National Cancer Institute (NCI);<br>Oregon State University; OHSU Knight Cancer Institute	University of Minnesota;<br>National Cancer Institute (NCI)	NYU Langone Health
Gender	All	All	Male	All	All
Age	18 Years and above	20 Years to 65 Years	21 Years and above	21 Years to 70 Years	Child, Adult, Older Adult
Phases	Phase 1	Phase 1, Phase 2	Not Applicable	Phase 2	Phase 1
Enrollments	100	55	98	107	No data
Study Type	Interventional	Interventional	Interventional	Interventional	Interventional
Study Designs	Allocation: Randomized	Allocation: N/A, Intervention Model: Single Group	Allocation: Randomized. Intervention Model: Parallel Assignment;	Allocation: Randomized;<br>Intervention Model: Crossover Assignment;<br>Masking: Double (Participant, Investigator);	Primary Purpose: Prevention

		Assignment, Masking: None (Open Label), Primary Purpose: Prevention	Masking: Triple (Participant, Care Provider, Investigator); Primary Purpose: Prevention	Primary Purpose: Prevention	
Completion Date	April 5, 2023	1/4/2014	1/12/2015	1/1/2013	No data
Locations	Roswell Park Cancer Institute, Buffalo, New York, United States	Georgetown Clinical Research Unit, Washington, District of Columbia, United States	OHSU Knight Cancer Institute, Portland, Oregon, United States. Portland VA Medical Center, Portland, Oregon, United States	Masonic Cancer Center, University of Minnesota, Minneapolis, Minnesota, United States	No data
URL	https://ClinicalTrials.gov/show/NCT04548193	https://ClinicalTrials.gov/show/NCT01790204	https://ClinicalTrials.gov/show/NCT01265953	https://ClinicalTrials.gov/show/NCT00691132	https://ClinicalTrials.gov/show/NCT00005883

Abbreviations: BSE—Broccoli sprout extract; HDAC6—Histone Deacetylase 6; ITCs—Isothiocynates; NCI—National Cancer Institute; NNK—4-(methylnitrosamino)-1-(3-pyridyl)-1-butanone; PEITC—Phenethyl Isothiocyanate; SFN—Safranal.
